# Identification of potential salivary biomarker panels for oral squamous cell carcinoma

**DOI:** 10.1038/s41598-021-82635-0

**Published:** 2021-02-09

**Authors:** Anu Jain, Chinmaya Narayana Kotimoole, Sushmita Ghoshal, Jaimanti Bakshi, Aditi Chatterjee, Thottethodi Subrahmanya Keshava Prasad, Arnab Pal

**Affiliations:** 1 Department of Biochemistry, Post Graduate Institute of Medical Education and Research, Chandigarh, 160012 India; 2 Centre for Systems Biology and Molecular Medicine, Yenepoya Research Centre, Yenepoya (Deemed To Be University), Mangalore, 575018 India; 3 Department of Radiotherapy, Post Graduate Institute of Medical Education and Research, Chandigarh, 160012 India; 4 Department of Otolaryngology, Post Graduate Institute of Medical Education and Research, Chandigarh, 160012 India; 5 Institute of Bioinformatics, International Technology Park, Bengaluru, 560066 India; 6 Manipal Academy of Higher Education (MAHE), Manipal, 576104 Karnataka India

**Keywords:** Oral cancer, Tumour biomarkers, Diagnostic markers

## Abstract

Oral squamous cell carcinoma (OSCC) is one of the most prevalent cancers worldwide with the maximum number of incidences and deaths reported from India. One of the major causes of poor survival rate associated with OSCC has been attributed to late presentation due to non-availability of a biomarker. Identification of early diagnostic biomarker will help in reducing the disease morbidity and mortality. We validated 12 salivary proteins using targeted proteomics, identified initially by relative quantification of salivary proteins on LC–MS, in OSCC patients and controls. Salivary AHSG (p = 0.0041**) and KRT6C (p = 0.002**) were upregulated in OSCC cases and AZGP1 (p ≤ 0.0001***), KLK1 (p = 0.006**) and BPIFB2 (p = 0.0061**) were downregulated. Regression modelling resulted in a significant risk prediction model (p < 0.0001***) consisting of AZGP1, AHSG and KRT6C for which ROC curve had AUC, sensitivity and specificity of 82.4%, 78% and 73.5% respectively for all OSCC cases and 87.9%, 87.5% and 73.5% respectively for late stage (T3/T4) OSCC. AZGP1, AHSG, KRT6C and BPIFB2 together resulted in ROC curve (p < 0.0001***) with AUC, sensitivity and specificity of 94%, 100% and 77.6% respectively for N0 cases while KRT6C and AZGP1 for N+ cases with ROC curve (p < 0.0001***) having AUC sensitivity and specificity of 76.8%, 73% and 69.4%. Our data aids in the identification of biomarker panels for the diagnosis of OSCC cases with a differential diagnosis between early and late-stage cases.

## Introduction

Oral cancer, with around 90% cases consisting of squamous cell type, is amongst the top ten prevalent (~ 0.6 million) cancers in males around the world^1,2^ with approximately 26% cases being reported from India alone. In India, it is one of the most prevalent (~ 0.2 million) cancer in males^1,2^. India has an incidence rate of around 0.1 million per year with around 70% mortality rate. Even with the advancement in treatment strategies in the last 2 decades, the survival rate of OSCC is still very poor which is often associated with the late presentation of the disease. Non-availability of a suitable tumour marker could be one of the major attributions towards this. Histopathological evaluation of tumour tissue biopsy along with radiological investigations are currently available diagnostic modality for oral cancer, which is an invasive procedure and advised once visible symptoms start to appear. The multistep, prolonged and invasive procedures of current confirmatory methods render it unsuitable as a screening tool. In this context, a non-invasive biomarker will be extremely useful for screening and early detection of the disease^3^.

One promising approach to identify the potential biomarkers is to analyse the cancer-related biomolecules in bodily fluids. Saliva, being the potential biofluid for surveillance of general health and diagnosis of disease and in the proximity of the oral cavity, makes a perfect biological fluid for identification of biomarker(s) for oral cancers^4,5^. In addition, the non-invasive procedure for collection of saliva makes salivary biomarkers ideal as a screening tool for oral squamous cell carcinoma (OSCC).

In this study, we identified potential biomarker panels of salivary proteins identified by estimating the levels of candidate salivary proteins using a targeted proteomics approach. We validated 12 candidate proteins (which were identified through Tandem Mass Tag (TMT) based relative quantification of the salivary proteome of OSCC, data not shown) using parallel reaction monitoring (targeted proteomics). Using this approach absolute quantification of candidate proteins was done in saliva resulting in the identification of potential biomarker panels with high sensitivity and specificity. REporting recommendations for tumour MARKer prognostic studies (REMARK) criteria was followed for reporting the study results^6^.

## Results

Among the recruited cases and controls 80% were males and 10% were females. The mean age of cases and controls was 54.6 years and 54 years respectively. Of the total recruited cases, 48% (n = 24) had no regional lymph node metastasis (N0) while 52% (n = 26) had regional lymph node metastasis (N+). 36% (n = 18) had a primary tumour of stage T1/T2 and 64% (n = 32) had a primary tumour of stage T3/T4. Post-treatment disease status was recorded for all cases. Only 44 cases could be followed-up to record the status and 6 were lost to follow-up. The status was recorded as NED (No Evidence of Disease) for patients who had no evidence of disease after treatment completion and progressive disease for cases which showed progressive disease after completion of treatment. Median follow-up time was 6 months. Ten cases were found to have no evidence of disease after treatment and 34 were having progressive disease.

### Five proteins were significantly dysregulated in OSCC cases

**Figure 1:**
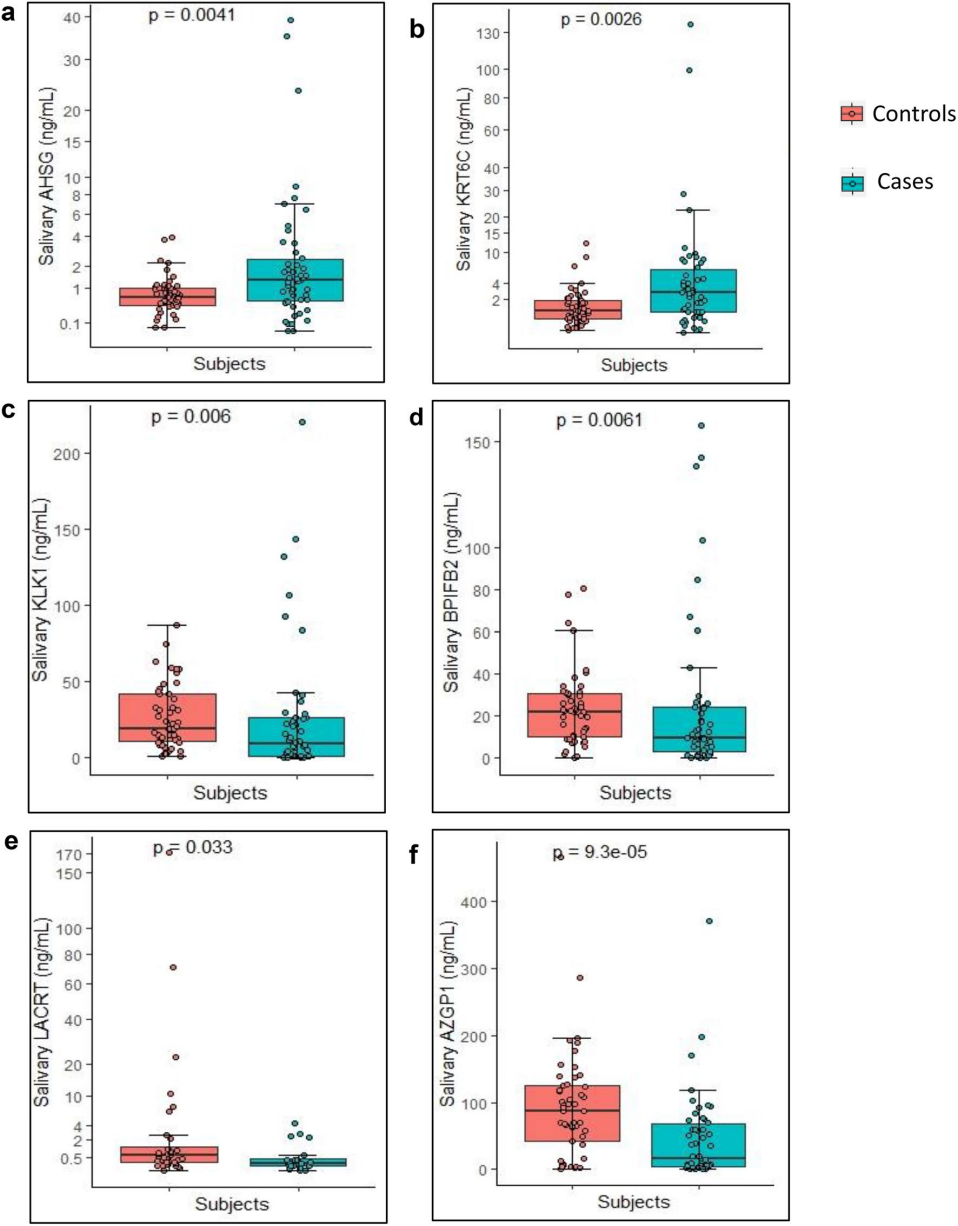
Levels of significantly dysregulated proteins in OSCC. Boxplot representing the levels of dysregulated proteins which were found to be significantly different between OSCC cases (n = 50) and healthy controls (n = 49) (Each dot represents individual value). Proteins (**a**) AHSG and (**b**) KRT6C were significantly upregulated with p value of 0.0041** and 0.0026** respectively. Proteins (**c**) KLK1, (**d**) BPIFB2, (**e**) LACRT and (**f**) AZGP1 were downregulated with p value of 0.006**, 0.0061**, 0.033* and < 0.0001***, respectively. Wilcoxon sum rank test was used to compare the median protein levels of controls and cases (p < 0.5 was the cut off for statistical significance). The levels of the proteins are represented in Table 1.

The salivary levels of the 12 candidate proteins are mentioned in Table 1. Out of the twelve proteins validated, two proteins AHSG and KRT6C were significantly upregulated and four proteins, AZGP1, KLK1, BPIFB2 and LACRT were found to be significantly downregulated (Fig. 1) (LACRT was not detected in all the cases and controls and was excluded in the further analysis).

**Table 1:** Median levels of protein along with interquartile range as quantified using PRM.

S. no.	Protein	Levels in controls (ng/ml)		Levels in cases (ng/ml)		p value
		Median	Interquartile range	Median	Interquartile range	
1	S100A7	1.96 (n = 46)	2.9–0.95	2.3 (n = 38)	11.16–1.02	0.22
2	*BPIFB2*	22.21 (n = 48)	31.08–10.69	9.6 (n = 50)	24.06–3.26	0.0061**
3	S100A9	12.24 (n = 47)	31.31–5.95	7.35 (n = 50)	19.34–1.63	0.071
4	CORO1A	0.45 (n = 48)	0.84–0.22	0.28 (n = 50)	1.02–0.1	0.42
5	**KRT6C**	0.93 (n = 49)	1.83–0.43	2.2 (n = 50)	5.19–0.7	0.002**
6	IGLL5	23.95 (n = 47)	39.16–14.44	23.42 (n = 50)	44.52–9.87	0.6
7	*KLK1*	19.39 (n = 49)	41.86–11.10	9.25 (n = 50)	26.24–1.19	0.006**
8	*LACRT*	0.6 (n = 32)	1.32–0.23	0.2 (n = 20)	0.43–0.13	0.033*
9	LCN2	5.91 (n = 49)	13.61–2.18	8.11 (n = 49)	25.88–2.12	0.44
10	PSAP	1.27 (n = 49)	1.68–0.85	1.2 (n = 49)	2.68–0.64	0.83
11	*AZGP1*	89.48 (n = 48)	125.04–47.06	16.87 (n = 50)	67.51–4.25	< 0.0001***
12	**AHSG**	0.65 (n = 49)	0.99–0.43	1.31 (n = 50)	2.40–0.57	0.0041**

p value reported was obtained using Wilcoxon Sum Rank test. Significantly upregulated proteins are highlighted in bold and significantly downregulated proteins are highlighted in italic.

Bold italics indicate statistical significance (p < 0.05).

**Figure 2:**
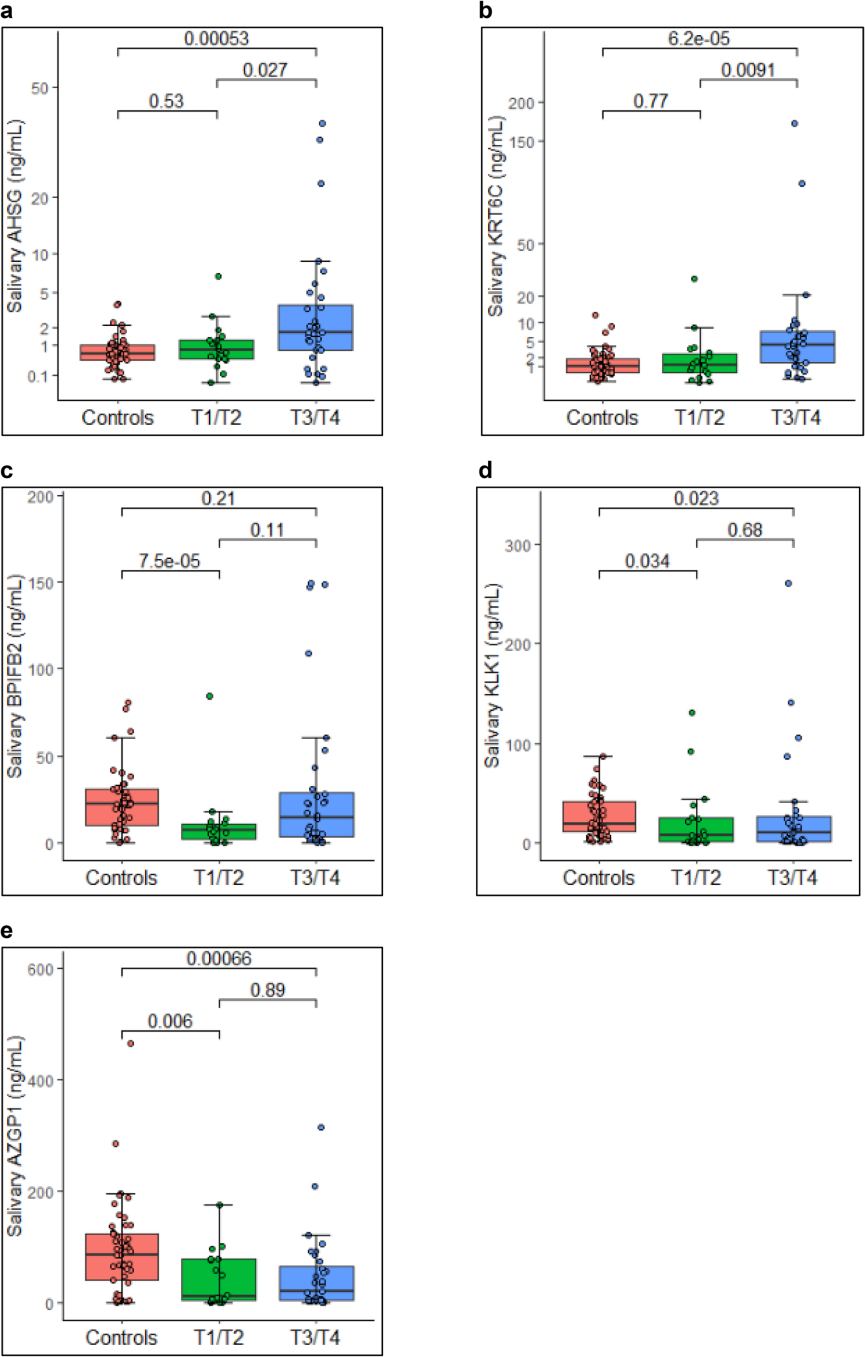
Levels of significantly dysregulated proteins as per primary tumour stage. Boxplot representing the levels of significantly dysregulated proteins amongst healthy controls (n = 49), T1/T2 stage OSCC (n = 18) and T3/T4 (n = 36) stage OSCC cases (Each dot represents individual value). Proteins (**a**) AHSG and (**b**) KRT6C were significantly upregulated in late stage cases. Proteins (**c**) BPIFB2 and (**d**) KLK1 were downregulated in both the stages while (**e**) AZGP1 was significantly downregulated in early stage cases. Wilcoxon sum rank test was used to compare the median protein levels of controls and cases (p < 0.5 was the cut off for statistical significance).

**Figure 3:**
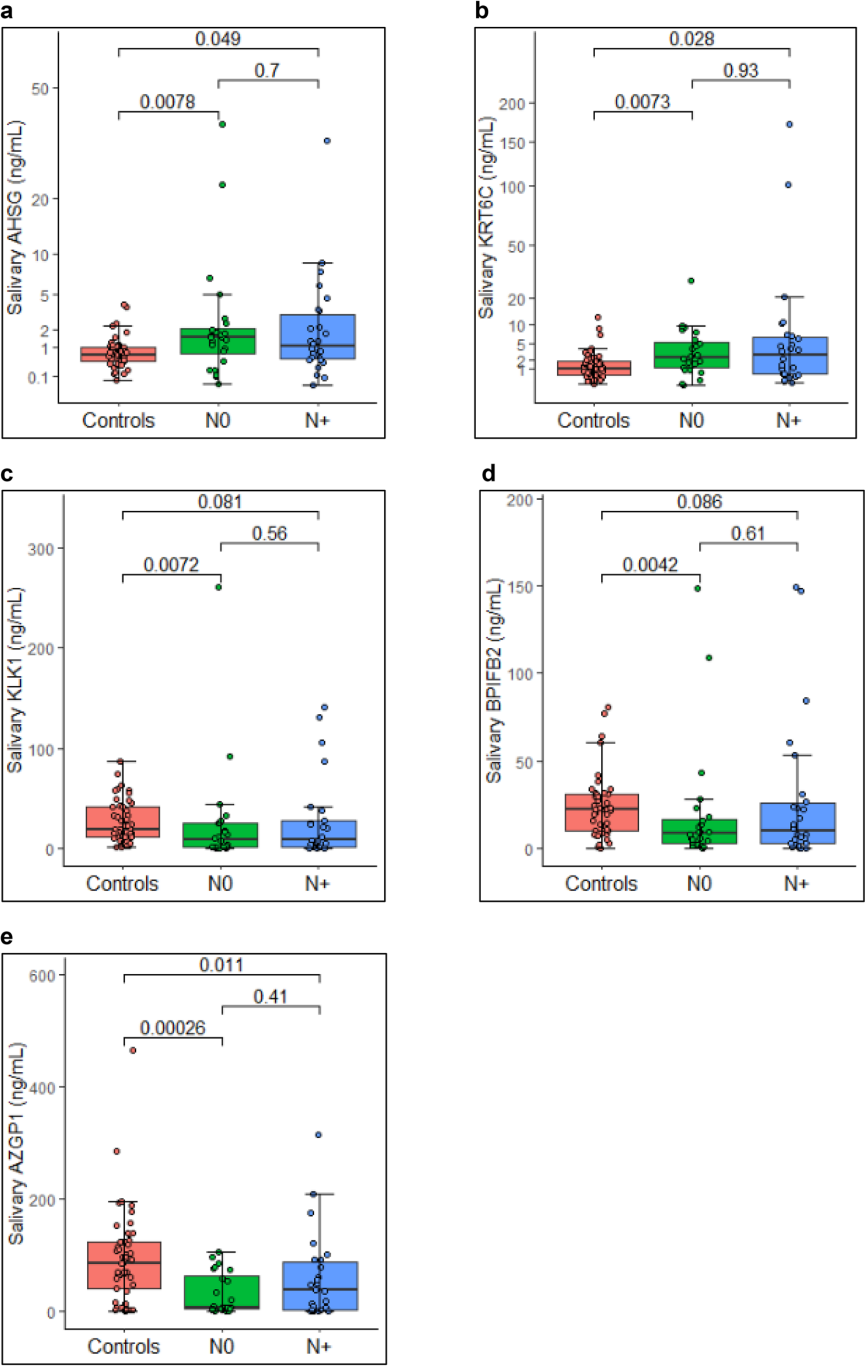
Levels of significantly dysregulated proteins as per regional lymph node metastasis. Boxplot representing the levels of significantly dysregulated proteins amongst healthy controls (n = 49), N0 stage OSCC (n = 24) and N+ (n = 26) stage OSCC cases (Each dot represents individual value). Proteins (**a**) AHSG and (**b**) KRT6C were significantly upregulated in late stage cases. Proteins (**c**) KLK1 and (**d**) BPIFB2 were downregulated in both the stages while (**e**) AZGP1 was significantly downregulated in early stage cases. Wilcoxon sum rank test was used to compare the median protein levels of controls and cases (p < 0.5 was the cut off for statistical significance).

Levels of the five significant proteins; AHSG, KRT6C, AZGP1, KLK1 and BPIFB2 were further analysed and compared as per disease stage. Figure 2 represents salivary levels of the proteins as per the primary tumour stage and Fig. 3 represents the salivary proteins level as per the regional lymph node metastasis. AHSG and KRT6C were significantly upregulated in T3/T4 stage while BPIFB2 was significantly downregulated in T1/T2 stage. AZGP1 and KLK1 were significantly downregulated in both the T1/T2 and T3/T4 stage. However, only AHSG and KRT6C represented a statistically significant difference between T1/T2 and T3/T4 stage. As per regional lymph node metastasis, AHSG, KRT6C (upregulated) and AZGP1 (downregulated) were significantly dysregulated in both N0 and N+ stage while BPIFB2 and KLK1 were significantly downregulated in N0 stage only. None of the proteins individually could be used as a marker to distinguish between N0 and N+ stage.

### Differential levels of significant proteins as per the tobacco consumption habits

**Figure 4:**
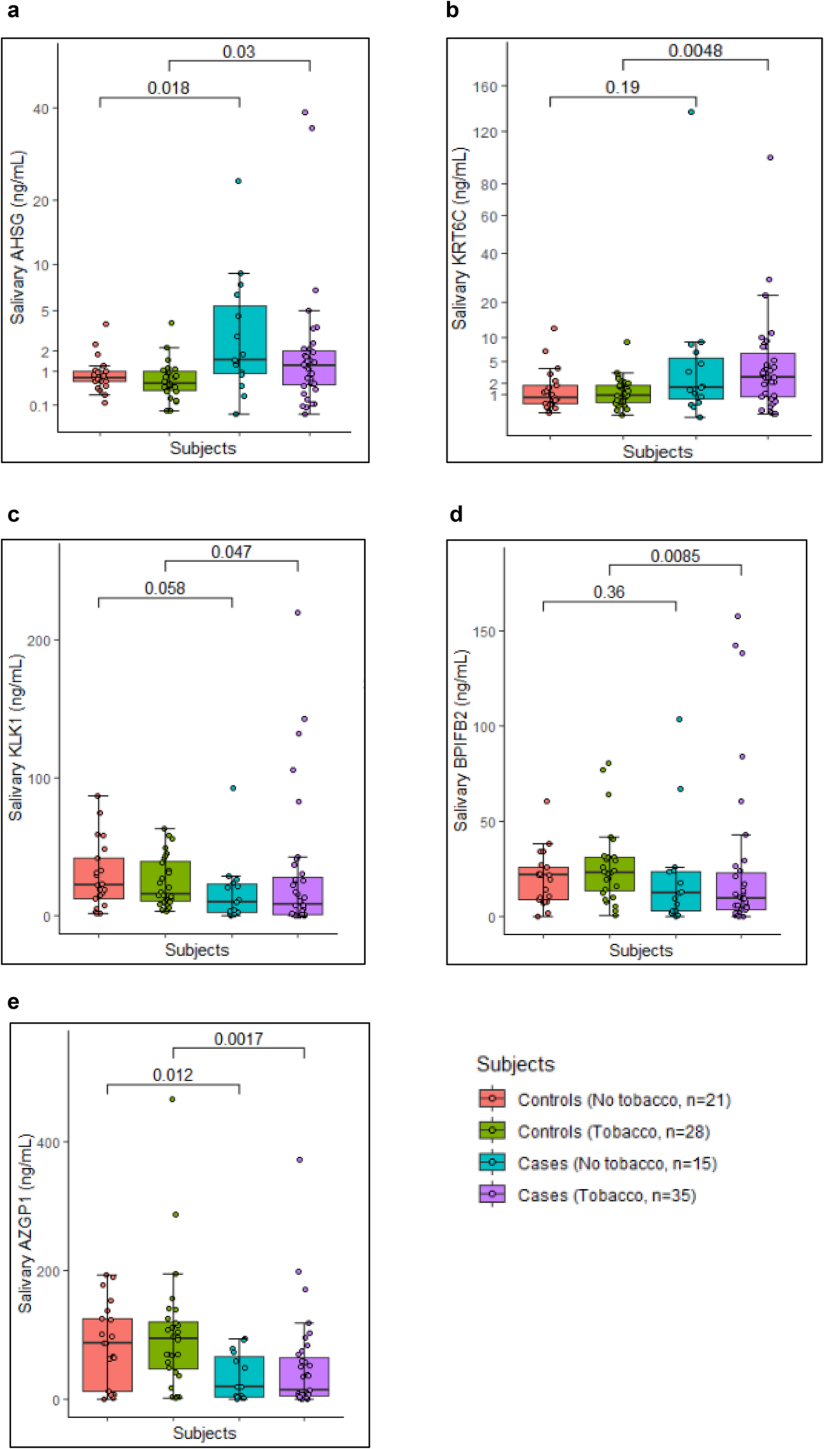
Levels of significantly dysregulated proteins as tobacco consumption habits of study subjects. Boxplot representing the levels of significantly dysregulated proteins amongst controls and OSCC cases with and without tobacco consumption habits. (**a**) AHSG was upregulated in cases compared to controls irrespective of the tobacco consumption habits (**b**) KRT6C was significantly upregulated in cases having tobacco consumption habits. Proteins (**c**) KLK1 and (**d**) BPIFB2 were downregulated in cases with tobacco consumption habits. (**e**) AZGP1 was significantly downregulated in cases irrespective of the tobacco consumption habits. Wilcoxon sum rank test was used to compare the median protein levels of controls and cases (p < 0.5 was the cut off for statistical significance).

Study subjects were classified into tobacco consumers and no tobacco consumers groups per their tobacco consumption habits. Twenty-eight individuals in control group had tobacco consumption habits in smoking or chewing form, while in case group 35 individuals had tobacco consumption habits. Rest of the individuals (total 50 cases and 49 controls) in both the groups did not have tobacco consumption habits in any form. Figure 4 represents the levels of significant proteins in study subjects as per tobacco consumption habits. AHSG and AZGP1 were dysregulated in cases compared to controls irrespective of their tobacco consumption habits. While KRT6C, KLK1 and BPIFB2 were significantly dysregulated only in the cases having tobacco consumption habits.

### Sensitivity and specificity of the significant protein

**Figure 5:**
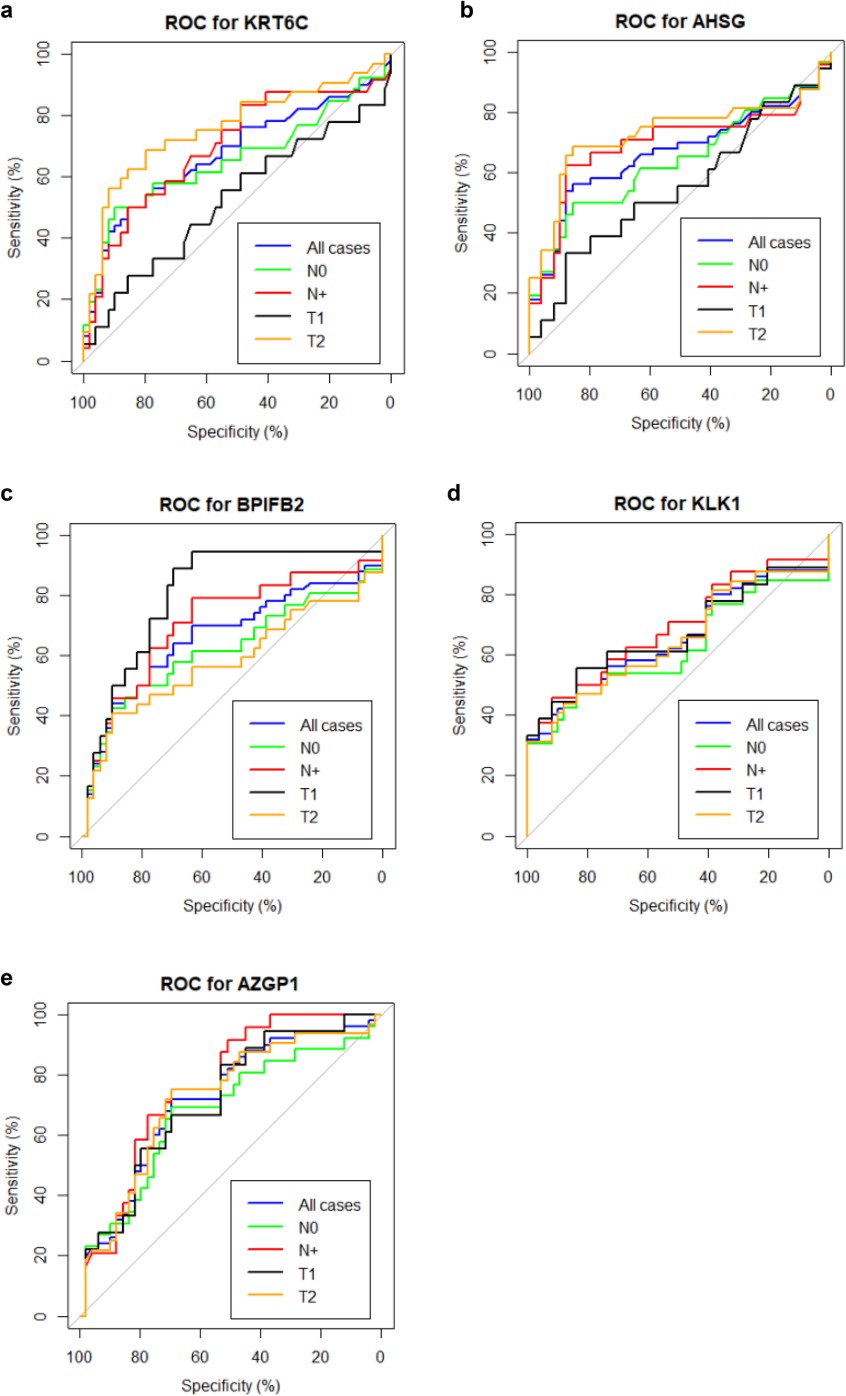
ROC curve for significantly dysregulated proteins (a) KRT6C (**b**) AHSG (**c**) BPIFB2 (**d**) KLK1 and (**e**) AHSG. Characteristics of the curves are mentioned in Table 2.

ROC curve was produced using the pROC package of R to observe the sensitivity and specificity of the significantly dysregulated proteins. ROC curve was plotted for the five significantly dysregulated proteins for all controls and all OSCC cases, controls and N0 OSCC cases, controls and N + OSCC cases, controls and T1 OSCC cases or controls and T2 OSCC cases (Fig. 5). The ROC curve of AZGP1 was significant for all conditions. ROC curve of AHSG and KRT6C was significant for all except the early stage of the primary tumour (T1/T2). For KLK1 ROC curve was significant for all conditions except N+ cases and for BPIFB2 curve was significant for all conditions except N+ cases and late stage of primary tumour cases (T3/T4) (Table 2).

**Table 2:** Characteristics of ROC curve for significantly dysregulated proteins.

	p value	AUC (%)	SEN (%)	SPE (%)	PPV (%)	NPV (%)
**AHSG**						
All cases	0.002**	66.80	60	69.39	62	69
T1	0.24	55.2	–	–	–	–
T2	< 0.0001***	72.9	68.8	85.7	75.9	80.8
N0	0.001**	69.3	62.5	87.8	71.4	82.7
N+	0.001**	63.9	50.0	85.7	65.0	76.4
**KRT6C**						
All cases	0.008**	64	60	63.26	64	63
T1	0.180	52.4	–	–	–	–
T2	< 0.0001***	75.8	68.8	79.6	68.8	79.6
N0	0.005**	69.5	50.0	85.7	63.2	77.8
N+	0.0009***	65.5	50.0	89.8	72.2	77.2
**KLK1**						
All cases	0.005**	66	58	69.39	58	69
T1	0.03*	67.0	55.6	83.7	55.6	83.7
T2	0.02*	64.9	31.3	98.0	100.0	69.0
N0	0.007**	69.3	45.8	91.8	73.3	77.6
N+	0.08	62.3	–	–	–	-
**BPIFB2**						
All cases	0.003**	66	70	64.58	72	65
T1	0.003**	80.5	88.9	69.4	51.6	94.4
T2	0.32	58.2	–	–	–	–
N0	0.004**	70.7	79.2	63.3	51.4	86.1
N+	0.08	62.1	–	–	–	–
**AZGP1**						
All cases	< 0.0001***	72.80	74	74.83	74	71
T1	0.004**	71.8	83.3	53.1	39.5	89.7
T2	0.004**	72.2	75.0	69.4	61.5	81
N0	< 0.0001***	76.5	75.0	69.4	54.5	85
N+	0.05*	67.9	69.2	69.4	54.5	81

*AUC* area under the curve, *SEN* sensitivity, *SPE* specificity, *PPV*: predictive value, *NPV* negative predictive value.

Bold values indicate statistical significance (p < 0.05).

### Logistic regression revealed a biomarker panel for diagnosis of OSCC cases

Multivariate logistic regression was applied to check the relation of protein levels on the disease status. Case and control were selected as the dependent variable and protein levels as the independent variable.

**Figure 6:**
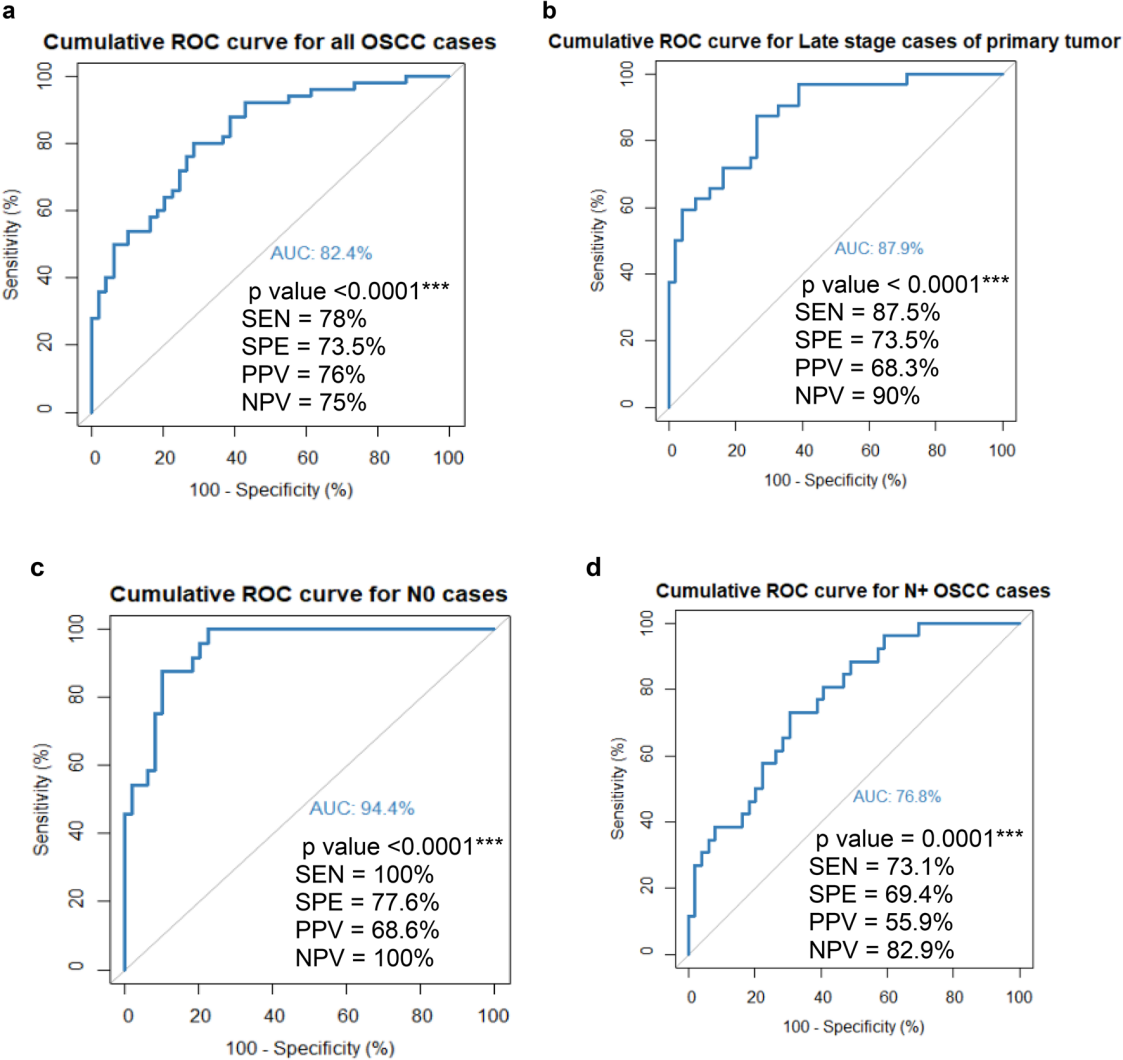
Cumulative ROC curve for the risk prediction models. (**a**) ROC curve for all the cases and controls for the model formed with the proteins AHSG, KRT6C and AZGP1 (**b**) ROC curve for the cases of late stage of primary tumour (T3/T4) and controls for the model formed with the proteins AHSG, KRT6C and AZGP1 (**c**) ROC curve for the cases with N0 stage and controls for the model formed with the proteins AHSG, KRT6C, AZGP1 and BPIFB2 (**d**) ROC curve for the cases with N+ stage and controls for the model formed with the proteins KRT6C and AZGP1. The characteristics of the ROC curve are mentioned in the inset of each figure. *AUC* area under the curve, *SEN* sensitivity, *SPE* specificity, *PPV* positive predictive value and *NPV* negative predictive value.

The analysis revealed that AHSG, KRT6C and AZGP1 together formed a risk prediction model for all the cases of OSCC (Table 3). The ROC curve plotted for this model was highly significant with a p value < 0.0001*** and area under the curve of 82.4% (Fig. 6a). The model was further found to be significant for late-stage cases of the primary tumour (T3/T4) (Table 3) with p value < 0.0001*** and area under the curve of 87.9% (Fig. 6b).

**Table 3:** Multiple regression analysis of the significant proteins for OSCC cases (All, Late T stage, N0 and N+).

	AHSG	KRT6C	AZGP1	BPIFB2	
p value	0.012*	0.03*	0.003**	ns	ALL OSCC cases
β coefficient	0.53	0.184	− 0.01	–	
Odds ratio	1.7	1.22	0.98	–	
Lower bound	1.21	1.01	0.97	–	
Upper bound	3.7	1.52	0.99	–	
p value	0.032*	0.006**	0.001**	ns	Late stage cases of primary tumor (T3/T4)
β coefficient	0.68	0.32	− 0.02	–	
Odds ratio	1.97	1.38	0.97	–	
Lower bound	1.17	1.12	0.95	–	
Upper bound	4.06	1.8	0.98	–	
p value	0.003**	0.006**	0.009**	0.003**	OSSC cases of N0 stage
β coefficient	1.96	0.492	− 0.038	− 0.12	
Odds ratio	7.14	1.63	0.96	0.88	
Lower bound	2.2	1.2	0.93	0.79	
Upper bound	35.5	2.4	0.9	0.94	
p value	–	0.02*	0.005**	ns	OSSC cases of N+ stage
β coefficient	–	0.27	− 0.011	–	
Odds ratio	–	1.31	0.98	–	
Lower bound	–	1.1	0.97	–	
Upper bound	–	1.63	0.99	–	

*Ns* non-significant.

Bold values indicate statistical significance (p < 0.05).

Further analysis revealed that AHSG, KRT6C, AZGP1 and BPIFB2 together formed a significant risk prediction model for N0 cases of OSCC (Table 3) and the ROC curve for this model was highly significant with p value < 0.0001*** and area under the curve of 94.4% (Fig. 6c). Finally, AZGP1 and KRT6C formed a significant model (*p* < 0.0001***) for OSCC cases of N+ stage (Table 3). The ROC curve of this model has an area under the curve of 76.8% (Fig. 6d).

### Survival analysis

Kaplan Meier analysis was done to analyse the impact of significant proteins on post-treatment disease status of the cases. However, the data obtained was not significant.

## Discussion

We executed this case–control cohort study with an aim to identify potential biomarker(s) for OSCC. We evaluated salivary proteins as a potential biomarker(s) for early diagnosis of the disease.

We used parallel reaction monitoring (targeted proteomics) approach which is promising in protein quantification and holds great clinical applications. Parallel reaction monitoring (PRM), where all transitions are analysed simultaneously in parallel, provides enhanced selectivity giving better results with lower limit of detection and quantification^7^. Using this highly sensitive analytical PRM approach 5 of the 12 validated candidate proteins were found to be significantly dysregulated in the saliva of patients studied.

AZGP1, BPIFB2 and KLK1 were significantly downregulated in our data of which role of KLK1 and AZGP1 is well reported in cancer but BPIFB2 remains unexplored.

AZGP1, an important protein involved in insulin sensitivity and plays a role in metabolism and cell cycle^8,9^, which are known to be altered in cancer progression^10,11^. Low mRNA/protein expression of AZGP1 is correlated with disease progression and poor survival in pancreatic cancer^12,13^. In our data, we have observed significantly low salivary levels of AZGP1 in OSCC cases compared to healthy controls. Different studies have reported contrasting expression of AZGP1 in different cancers^14–19^. Ibrahim et. al reported low RNA levels of AZGP1 in OSCC tumour tissue of betel quid users^20^ which supports our observation at the protein level as well. Role of AZGP1 in the suppression of cellular invasion and migration^21–23^, suggests its association with poor disease response. However, the reduction in levels of AZGP1 in cancer patients mandates a very sensitive detection method for it to be successful as a tumour marker in clinical practice. In few studies high expression of AZGP1 was reported in case of smokers and it is linked to the metabolic dysregulation resulting in fat loss in smokers^24,25^. However, in our data we could not find much difference in AZGP1 levels in amongst cases and controls group based on their tobacco consumption habits. So, the expression analysis of AZGP1 in two cases, smoking and cancer where one is the cause and the other is the effect, could not be correlated from our data. This may be attributed to relatively small number of samples included in our study.

BPIFB2, a member of the lipid transfer/lipopolysaccharide binding protein family. The protein is not much explored in cancer. BPIFB2 mRNA expression was reported to be dysregulated in OSCC tumours compared to the normal counterparts^26^. Our observations are also in agreement with reported literature. Statistically significant downregulation of BPIFB2 was observed in the early stage OSCC (both primary tumour stage and regional lymph node metastasis).

KLK1, a member of the serine protease protein family, is involved in many physiological functions like the remodelling of the extracellular matrix, cellular proliferation and differentiation, angiogenesis, apoptosis. We observed low expression of KLK1 in OSCC as compared to healthy controls which are supported by literature where expression of KLK1 was found to be downregulated in multiple cancers including head and neck cancers of which oral cancer is a part^27^.

In our data sets, AHSG and KRT6C were observed to be significantly upregulated in OSCC. KRT6C is a subtype of type II keratin and has its expression restricted to distinct epithelial type, like filiform papillae of the tongue, the stratified epithelial lining of the oesophagus and oral mucosa and in glandular epithelia^28–30^. Expression of KRT6C is associated with abnormal differentiation or enhanced proliferation, like in case of wound healing or cancer with exception of only a few body sites^31,32^. This supports our observation that the expression of this protein was significantly upregulated in the late stage of the primary tumour (T3/T4). The Cancer Genome Atlas (TCGA) also reports high RNA expression of KRT6C in head and neck cancer. When compared in terms of tobacco consumption history of the study population, we observed that there was a significant difference in salivary KRT6C levels between cases and controls with tobacco consumption habits but not between cases and controls without tobacco consumption habits. In the discovery phase data also the expression of the protein was found to be upregulated with other keratins in OSCC cohort with tobacco chewing habits. This observation supports the fact that tobacco consumption causes molecular alterations in the cell which progresses to cancer and is also suggestive of association of keratins with tobacco induced OSCC.

AHSG is a protein of cystatin superfamily with multiorgan expression during embryogenesis^33^. However, in adults AHSG expression limits mainly to the liver and in some cases to osteoblast^34^. It is a multifunctional protein^35^ reported to be associated with various disease conditions^36–39^ including cancer^40,41^.

However, few studies are reporting the role of salivary AHSG in diseases like periodontal disease, obstructive sleep apnea etc.^42,43^. We report here for the first time, upregulated salivary AHSG levels in oral cancer (in comparison to healthy controls). AHSG expression levels were almost twice in cancer cases compared to the controls. This observation was consistent when analysed using three different approaches -global proteomics, targeted proteomics and ELISA.

We observed a significant difference in AHSG and KRT6C expression between controls and late stage OSCC cases (T3/T4 stage of the primary tumour) suggesting the role of these proteins in disease progression towards the aggressive course and this is supported by reported observations that AHSG is required for cellular adhesion, proliferation, migration and invasion of cancer cells^44–47^. KRT6C expression is also associated with abnormal and enhanced proliferation^48^.

Expression analysis as per the tobacco consumption revealed that KLK1, KRT6C and BPIFB2 were dysregulated with statistical significance in OSCC cases having tobacco consumption habits only. However, due to small number of study subjects in tobacco consumer and non-consumer group, we could not derive a significant conclusion with this analysis.

Since cancer is a multifactorial disease, we further used multivariate logistic regression modelling strategy to check the cumulative effect of these proteins compared to their individual effect. We found that AHSG, KRT6C and AZGP1 together form a highly sensitive prediction model with high sensitivity, specificity and accuracy which is better than their individual diagnostic potential as the average AUC, sensitivity and specificity of individual proteins increased from 66%, 64% and 67–83%, 78% and 73%, respectively. These results indicate that AHSG, KRT6C and AZGP1 together can serve as a potential biomarker panel for diagnosis of oral cancer. The ROC curve for this panel was statistically significant for late stage OSCC cases (T3/T4) compared to early stage OSCC cases (T1/T2). Another panel consisting of proteins AHSG, KRT6C, BPIFB2 and AZGP1 were statistically significant for OSCC cases of N0 stage and non-significant for N+ stage while for OSCC cases diagnosed with N+ stage AZGP1 and KRT6C resulted in statistically significant panel indicating that this can be useful for differential diagnosis of regional lymph node metastasis.

Multivariable biomarker panel approach has been reported to be better in terms of accuracy, sensitivity and specificity, not just for oral cancer but other pathological conditions^49,50^. The panel developed in our study shows better diagnostic accuracy in comparison to the individual proteins.

As an outcome of this study, we report a sensitive biomarker panel which can be developed into a multiparameter rapid testing kit to explore its potential in clinical settings. However, the study being a pilot study in nature, the outcome needs to be evaluated in a larger patient population. For future directions, cross-validation of this prediction model in terms of accuracy, precision, sensitivity, specificity and positive and negative predictive values using a separate large cohort of OSCC cases, disease controls and healthy controls is needed so that the potential value of this prediction model as a biomarker panel in the clinical setting can be explored and a rapid detection kit can be developed for the model to facilitate population screening.

## Materials and methods

### Subjects

The study was approved by the Institutional Ethics Committee (approval No/PGI/IEC/2016/3397 dated 06-09-2016) at Post Graduate Institute of Medical Education and Research, Chandigarh. A prospective case–control study was designed. Patients attending the Department of Radiotherapy and Department of Otolaryngology at Post Graduate Institute of Medical Education and Research, Chandigarh (India), undergoing surgery and/or receiving the standard radio/chemo-therapy with curative intent based on disease stage, decided as per the approved clinical protocol in the institute were enrolled in the study. Fifty, biopsy proven OSCC cases and age and gender-matched 49 healthy volunteers were recruited after obtaining written informed consent and following the inclusion and exclusion criteria (supplementary data). In accordance with the approved protocol by the ethics committee and without following any invasive procedure, unstimulated saliva samples (at least 5 ml of saliva) were collected, following at least half an hour abstinence from any food and fluid including water, by collecting the saliva directly in a 50 ml centrifuge tube. The collected sample was centrifuged at 5000 rpm for 20 min at 4 °C and supernatant were collected and preserved at − 80 °C for further analysis. All the experiments were performed following the relevant guidelines and the approved protocol by the Institutional Ethics Committee. Patients were followed up after treatment completion until the end of the study or till the event (progressive disease) was recorded.

### Selection of dysregulated proteins as a potential candidate for biomarkers

The candidate proteins were selected from a preliminary shotgun proteomic data obtained by TMT tag-based relative quantification of salivary proteins of OSCC cases on LC–MS (data not presented) where 135 dysregulated salivary proteins (supplementary Table S1) were identified. These proteins were analysed for their gene ontology, protein–protein interaction network and fold change to select the candidate proteins. With this strategy, 12 highly dysregulated proteins (Table 1), also reported to play a significant role in cancer biology were selected for further analysis by Parallel reaction monitoring (PRM) based absolute quantification on a mass spectrometer.

### Standard reference peptides for parallel reaction monitoring (PRM)

Quantotypic unique peptides (supplementary data) were chosen corresponding to the candidate proteins following the selection criteria for peptides for PRM. Tryptic peptides were purchased in the lyophilized form from JPT Peptide Technology (Berlin, Germany) in both light version and labelled version, where C terminal amino acid (lysine or arginine) was heavy labelled (K* = Lys U-^13^C_6_; U-^15^N_2_, R* = Arg U-^13^C_6_; U-^15^N_4_). Peptides were reconstituted as per the manufacturer’s instructions to a final concentration of 100 pmol/µl and serially diluted ranging from 256 to 0.5 fmol/µl to obtain ten working standard concentrations.

### Sample preparation for parallel reaction monitoring (PRM)

Total protein in the saliva samples was quantified using BCA Protein Assay Kit (#23227, Pierce Biotechnology, Rockford, USA) and following the manufacturer’s protocol. 50 µg of total protein from each sample was prepared for absolute quantification. Total protein was reduced, alkylated and trypsin digested. Digested samples were desalted using Sep-pak C18 cartridge (Waters), dried and reconstituted at the time of analysis with 0.1% formic acid and spiked in with the heavy labelled peptides with a concentration more than the limit of quantification as determined by the standard curves. (This section is mentioned in details in the supplementary data).

### PRM method: sample acquisition and data analysis

PRM method was developed using a pool of reference peptide to achieve good resolution and ion abundance. The method development and analysis part are mentioned in the supplementary data in details. Briefly, a 40 min liquid chromatography method was developed to resolve the peptides and a two-step mass spectrometer method was set to analyse the eluting peptides. First, a full scan MS was done to identify the precursor masses followed by a targeted MS of the selected precursor ions which were analysed and recorded on the orbitrap analyser.

The raw files were imported into the Skyline software to analyse and obtain the product ion transition area of each peptide precursor. A standard curve with the reference peptide pool was generated to calculate the limit of detection and quantification which was used as a reference to spike the heavy peptide concentration in the sample digest (Supplementary data). The ratio of light to the heavy summed transition area was multiplied with the amount of heavy peptide spiked in for the quantification of respective peptides in the samples. The samples were obtained in triplicate and were averaged for final quantification.

### Statistical analysis

R was used for the graphical presentation and statistical analysis of the data^51^. Shapiro Wilk normality test was used to check the normality distribution of the data and observing the non-gaussian distribution, Wilcoxon Sum Rank test was used to compare the median protein levels between two groups. Receiver Operating Characteristic (ROC) curve was generated to find out the optimum sensitivity, specificity and cut-off levels of proteins. Multivariate logistic regression was done to analyse the cumulative diagnostic potential of the proteins.

## Supplementary Information

Supplementary Information 1.

Supplementary Information 2.

## Data Availability

The proteomics data generated for the study is submitted to Panorama Public and can be accessed with the URL https://panoramaweb.org/APoscc.url. Alternatively, the data can be accessed http://www.proteomexchange.org using PXD020263 as the data identifier ID.
