# Supplementary material for: Identification of potential salivary biomarker panels for oral squamous cell carcinoma
Source: Sci Rep. 2021 Feb 9;11:3365. doi: 10.1038/s41598-021-82635-0 (PMC7873065; doi:10.1038/s41598-021-82635-0)
Supplement: Supplementary file 2 — Supplementary Information 2. [file 41598_2021_82635_MOESM2_ESM.docx]

**Title**

Identification of potential salivary biomarker panels for Oral Squamous Cell Carcinoma.

Anu Jain^1^, Chinmaya Narayana Kotimoole^2^, Sushmita Ghoshal^3^, Jaimanti Bakshi^4^, Aditi Chatterjee^5^, Thottethodi Subrahmanya Keshav Prasad^6^, Arnab Pal^7^*

1 Department of Biochemistry, Post Graduate Institute of Medical Education and Research, Chandigarh, 160012, India. [jainanu1291@gmail.com](mailto:jainanu1291@gmail.com)

2 Centre for Systems Biology and Molecular Medicine, Yenepoya Research Centre, Yenepoya (Deemed to be University), Mangalore, 575018, India. [chinnu.kemmaai@gmail.com](mailto:chinnu.kemmaai@gmail.com)

3 Department of Radiotherapy, Post Graduate Institute of Medical Education and Research, Chandigarh, 160012, India. [rtsushmita@gmail.com](mailto:rtsushmita@gmail.com)

4 Department of Otolaryngology, Post Graduate Institute of Medical Education and Research, Chandigarh, 160012, India. [drjayabakshi@gmail.com](mailto:drjayabakshi@gmail.com)

5 Institute of Bioinformatics, International Technology Park, Bangalore, 560066, India. [aditixchatterjee@gmail.com](mailto:aditixchatterjee@gmail.com)

6 Centre for Systems Biology and Molecular Medicine, Yenepoya Research Centre, Yenepoya (Deemed to be University), Mangalore, 575018, India. [keshav@yenepoya.edu.in](mailto:keshav@yenepoya.edu.in)

7 Department of Biochemistry, Post Graduate Institute of Medical Education and Research, Chandigarh, 160012, India. [pal.arnab@pgimer.edu.in](mailto:pal.arnab@pgimer.edu.in), [drarnabpal@gmail.com](mailto:drarnabpal@gmail.com) Phone no: +91-9530801817, +91-172-2755177

Corresponding author*

**Supplementary table S1 (Excel file)**

Table represents the dysregulated proteins in OSCC cohorts (compared to controls) identified upon shotgun proteomics. Column A, B and C represents the accession number, gene symbol and description of dysregulated proteins. Column D, E, F and G represents the fold change of the respective proteins in OSCC cohorts compared to controls. The fold change was calculated as a ratio of intensity of TMT ions in OSCC cohorts (smoker, chewer, smoker chewer and no risk) to that of the control cohort.

**Inclusion and exclusion criteria for the enrolment of patients and controls**

Biopsy proven cases of oral squamous cell carcinoma (OSCC) with age more than 18 years and willing to sign informed consent form were included in the study. Patients receiving any prior treatment for OSCC or presenting with co-morbidities or not willing to sign the consent form were not included. Patients were examined for dental or other oral diseases before start of the treatment and patients having any such infectious or inflammatory disorder were not included in the study.

Age and gender matched healthy individuals without any signs and symptoms of OSCC or other morbidities, with age more than 18 years willing to participate in the study and sign informed consent form were enrolled in the study.

**Reconstitution of the peptides**

Reference peptides were reconstituted with a solvent containing 80% of 0.1M ammonium bicarbonate and 20% of acetonitrile (ACN) (as per the manufacturer’s instruction) in a final concentration of 100 pmoles/µL to prepare a stock solution. The peptides selected for each protein is mentioned in the supplementary table S2.

**Supplementary table S2**: list of peptides selected for quantification using parallel reaction monitoring

| S.no. | Gene Symbol | Protein | Peptides selected |
| --- | --- | --- | --- |
| 1 | S100A7 | Psoriasin S100 calcium-binding protein A7 | GTNYLADVFEK |
| 2 | BPIFB2 | BPI fold-containing family B member 2 | AALSYVSEIGK |
| 3 | S100A9 | S100 calcium-binding protein A9 | DLQNFLK |
| 4 | CORO1A | Coronin 1A | LQATVQELQK and DAGPLLISLK |
| 5 | KRT6C | keratin, type II cytoskeletal 6C | TAAENEFVTLK |
| 6 | IGLL5 | immunoglobulin lambda-like polypeptide 5 | VTVLGQPK |
| 7 | KLK1 | Kallikrein 1 | LTEPADTITDAVK |
| 8 | LACRT | Lacritin | SILLTEQALAK |
| 9 | LCN2 | Lipocalin 2 | SYPGLTSYLVR |
| 10 | PSAP | Prosaposin A | QEILAALEK |
| 11 | AZGP1 | zinc-alpha-2-glycoprotein | AGEVQEPELR |
| 12 | AHSG | alpha-2-HS-glycoprotein | FSVVYAK |

**Sample preparation for Parallel Reaction Monitoring**

Total protein in the saliva samples was quantified using BCA Protein Assay Kit (#23227, Pierce Biotechnology, Rockford, USA) and following the manufacturer’s protocol. 50 µg of total protein from each sample was prepared for absolute quantification. Total protein was reduced by adding 100 mM dithiothreitol (DTT, #D0632-25G from Sigma-Aldrich, MO, USA) to a final concentration of 5 mM followed by incubation at 60°C for 60 minutes. Cysteine residues were irreversibly carbamidomethylated by adding 100 mM of iodoacetamide (IAA, #I1149-25G from Sigma-Aldrich, MO, USA) to a final concentration of 20 mM and incubating at room temperature for 10 minutes. Sequencing grade modified trypsin (#V5111, Promega, Madison, USA) was added in a ratio of 1:20 (trypsin: total protein) and incubated overnight at 37°C. Digestion reaction was stopped by acidifying the reaction mixture using 0.1% formic acid (#94318-250ML-F, Fluka). Digested samples were dried in a SpeedVac and reconstituted in 0.1% formic acid (FA) for desalting. Sep-pak C18 cartridge (Waters) was used for desalting the samples. Cartridge was activated with 100% ACN and equilibrated twice with 0.1% FA. After this the sample was coupled to the matrix by repeated cycles of sample loading. The cartridge was then washed twice with 0.1% FA. Finally, the sample was eluted in 50% ACN, 0.1% FA by 2 cycles of aspirating and dispensing. The desalted samples were dried in SpeedVac and reconstituted at the time of analysis with 0.1% formic acid (at a concentration of 1 µg/µL) and spiked in with the heavy labelled peptides with a concentration more than the limit of quantification as determined by the standard curves.

**Parallel Reaction Monitoring (PRM) method development and sample acquisition**

PRM analysis was done on Thermo Scientific Orbitrap Fusion Tribrid Mass Spectrometer (Thermo Scientific, Bremen, Germany) interfaced with Easy-nLC 1200 nanoflow liquid chromatography (Thermo Scientific, Odense, Denmark). Initially, pool of all quantotypic peptides in aqueous (0.1% formic acid) were acquired in Full MS scan mode to check their peptide precursor mass. All peptide precursor masses (m/z) identified with good abundance were included for PRM acquisition. Both method development and sample acquisition were performed with Acclaim PepMap 100 trap column (Thermo Scientific 75 µm x 2 cm, nanoViper, C18, 3 µm, 100Å) connected in tandem with PepMap RSLC C18 (Thermo Scientific, 3 µm, 100 Å, 75 µm × 15 cm) analytical column.

All 10 standard concentrations (256, 128, 64, 32, 16, 8, 4, 2, 1 and 0.5 fmol/µL) of quantotypic peptides were prepared in aqueous (0.1% formic acid) by serial diluting the concentration of light peptides. Heavy labelled peptides were spiked to each standard conc. in 50 fmol/µL as an internal standard (IS). These standards prepared were used to develop and standardize PRM method by varying LC gradient and acquisition parameters to acquire minimum 8-10 data points for each peptide and standard curve with regression coefficient (R^2^) of ≥ 0.99. Skyline was used to generate the calibration curve (CC) and calculate R^2^. To check the accuracy and R^2^ of the same peptides in saliva matrice as background, reverse calibration curve concept was followed. Where, heavy peptide concentrations were varied unlike aqueous calibration curve as endogenous light peptides would be already present in control saliva samples. However, heavy peptides (concentration of each peptide spiked in the sample digest is mentioned in supplementary table S3) were spiked for the acquisition of control and disease salivary protein digest to calculate the area ratio. In order to remove variations of peptide abundance in individual salivary protein digest samples and bring homogeneity, a pool of 25 controls salivary protein digest samples were used to generate the calibration curve.

Total 10 standards were prepared for the generation of calibration curve for each peptide. However, depending on the response and ion intensity of the standards and reproducibility across the replicates, atleast 6 best standards were chosen to generate the calibration curve.

Finally, a LC-MS-PRM method of 40 min was finalized with gradient LC method. Mobile phase A (0.1% formic acid in water) and Mobile phase B (0.1% formic acid in 80% acetonitrile) were used to load, separate and elute peptides at a flow rate of 250 nL/min. Mobile phase B% was set at 5% between 0-2 min and gradually increased to 40% at 32 min in a linear gradient fashion. Later % of B was increased to 100% at 34 min and maintained till 38 min to elute any highly hydrophobic particles, followed by decreasing it back to 5% at 39 min and maintained till 40 min. Injection volume of 1µL was set for all standards and samples throughout the study.

**Supplementary table S3:** Limit of quantification (fmoles/µL) and amount of heavy labelled peptides (fmoles/µL) spiked in the samples for absolute quantification.

| S. No. | Peptide | Protein | Limit of quantification (fmoles/µL) | Amount of peptide spiked in the samples (fmoles/µL) |
| --- | --- | --- | --- | --- |
| 1 | GTNYLADVFEK | S100A7 | 1 | 2 |
| 2 | AALSYVSEIGK | BPIFB2 | 0.5 | 2 |
| 3 | DLQNFLK | S100A9 | 0.5 | 2 |
| 4 | LQATVQELQK | CORO1A | 0.5 | 16 |
| 5 | DAGPLLISLK | CORO1A | 0.5 | 2 |
| 6 | TAAENEFVTLK | KRT6C | 0.5 | 4 |
| 7 | VTVLGQPK | IGLL5 | 0.5 | 2 |
| 8 | LTEPADTITDAVK | KLK1 | 0.5 | 2 |
| 9 | SILLTEQALAK | LACRT | 0.5 | 2 |
| 10 | SYPGLTSYLVR | LCN2 | 1 | 4 |
| 11 | QEILAALEK | PSAP | 0.5 | 2 |
| 12 | AGEVQEPELR | AZGP1 | 0.5 | 2 |
| 13 | FSVVYAK | AHSG | 0.5 | 32 |

The Orbitrap Fusion method consists of two experiments, i.e., a Full MS scan followed by PRM acquisition. Precursor masses were ionized in positive ionization mode. EASY Spray source with nano-electrospray ionization (NSI) technique, where spray voltage of 2.1 kV was set with ion transfer tube temperature of 275 °C. Full MS scan performed to identify peptide precursor masses ranging between 400-1500 m/z at a mass resolution of 60,000. The AGC target was set to 200,000 with maximum injection time (IT) of 20 ms. Post Full MS scan, a targeted MS2 (tMS^2^) was carried out for all precursors in the inclusion list. Peptide precursor masses were filtered in quadrupole mass analyzer with an isolation window width of 2.1 Da. Peptide precursors were fragmented by High collision energy induced dissociation (HCD) technique, where 35% collision energy applied with stepped collision energy of 5%. Fragment ions ranging between 100-2000 m/z were detected in Orbitrap at a mass resolution of 15,000. The AGC target for MS/MS scan was set to 50,000 with maximum IT of 50 ms. Both Full MS and tMS^2^ scans were acquired in profile data type. Transition list used for the targeted MS is given in the supplementary table S4, where we have fixed a retention time (RT) window of ±2 min for tMS^2^ of each precursor masses. Samples were analysed in triplicated and replicated value were averaged to obtain a single value.

**Supplementary table S4**: transition list included in the targeted method for the peptide quantification.

| m/z | charge | t start (min) | t stop (min) | Compound Name |
| --- | --- | --- | --- | --- |
| 421.2607 | 2 | 17.42 | 19.42 | VTVLGQPK |
| 425.2676 | 2 | 17.42 | 19.42 | VTVLGQPk |
| 564.2889 | 2 | 17.62 | 19.62 | AGEVQEPELR |
| 569.2928 | 2 | 17.62 | 19.62 | AGEVQEPELr |
| 579.33 | 2 | 17.71 | 19.71 | LQATVQELQK |
| 583.3367 | 2 | 17.71 | 19.71 | LQATVQELQk |
| 407.229 | 2 | 17.91 | 19.91 | FSVVYAK |
| 411.2357 | 2 | 17.91 | 19.91 | FSVVYAk |
| 611.8196 | 2 | 19.15 | 21.15 | TAAENEFVTLK |
| 615.826 | 2 | 19.15 | 21.15 | TAAENEFVTLk |
| 687.3605 | 2 | 19.41 | 21.41 | LTEPADTITDAVK |
| 691.3672 | 2 | 19.41 | 21.41 | LTEPADTITDAVk |
| 569.3116 | 2 | 19.65 | 21.65 | AALSYVSEIGK |
| 573.3185 | 2 | 19.65 | 21.65 | AALSYVSEIGk |
| 507.7957 | 2 | 20.16 | 22.16 | QEILAALEK |
| 511.802 | 2 | 20.16 | 22.16 | QEILAALEk |
| 593.8567 | 2 | 20.32 | 22.32 | SILLTEQALAK |
| 597.8638 | 2 | 20.32 | 22.32 | SILLTEQALAk |
| 439.2432 | 2 | 21.61 | 23.61 | DLQNFLK |
| 443.25 | 2 | 21.61 | 23.61 | DLQNFLk |
| 628.3382 | 2 | 22.74 | 24.74 | SYPGLTSYLVR |
| 633.3417 | 2 | 22.74 | 24.74 | SYPGLTSYLVr |
| 628.8115 | 2 | 23.39 | 25.39 | GTNYLADVFEK |
| 632.8187 | 2 | 23.39 | 25.39 | GTNYLADVFEk |
| 513.8137 | 2 | 23.68 | 25.68 | DAGPLLISLK |
| 517.8207 | 2 | 23.68 | 25.68 | DAGPLLISLk |

**Data analysis**

Skyline software (v19.1) was used for the data analysis. Peptide sequences which were selected for quantification were imported into the Skyline document. In the transition settings precursor charge 2 and ion type as y ions were selected in the filter tab. In the full scan tab isotope peaks were set to count and precursor mass analyzer was set to orbitrap at a resolution of 60,000 in the MS1 filtering section. In the MS/MS filtering section acquisition method was set to targeted and product mass analyzer as orbitrap at a resolution of 15,000 (At 200 m/z). All matching scans were included in the analysis. In the peptide settings, trypsin was selected as the enzyme in the digestion tab with 0 Max missed cleavages. In the modification tab, carbamidomethyl was selected as the structural modification, heavy isotope label type was selected.

**Determination of limit of quantification (LOQ)**

For standard curve, light was selected as the internal standard type. All peaks were inspected manually and top intense consistent ions were selected for quantification. Calibration curve was viewed after selecting the top intense and consistent ion peaks and selecting the product ions (same ions were selected for a peptide for all the samples) (Table S6, Figure S1). For calculation of limit of detection linear regression fit was selected with 1/x^2^ weightage, in the quantification tab in the Skyline software. Ratio to light was selected as the normalization method. Blank+(3*SD) was selected for calculation of limit of detection (LOD), where SD is the standard deviation of the y intercepts. Limit of quantification (LOQ) was determined by software.

**Quantification in samples**

Similar software settings were used for analysis of samples except the internal standard type was set to heavy now. Transition areas as observed in Skyline for each peptide was exported to MSexcel. Ratio of sum transition area of light peptide to sum transition area of heavy isotope labelled peptide was multiplied with the concentration of the heavy isotope labelled peptide spiked in the sample to obtain the absolute quantification of the peptides in the samples. For 11 proteins one peptide each protein was selected and for protein CORO1A two peptides were selected and average quantification obtained from both the peptides was considered as quantity of CORO1A in the samples. All the samples were acquired in triplicate and triplicate values were averaged for final quantification.

**Supplementary table S6**: list of standard concentrations and heavy to light area ratio obtained for each peptide.

| FSVVYAK for AHSG | | |
| --- | --- | --- |
| **Standard** | **Standard Concentration (fmol/µL)** | **Heavy to Light area ratio** |
| 1 | 0.5 | 1.34 |
| 2 | 1 | 2.53 |
| 3 | 2 | 4.81 |
| 4 | 4 | 9.83 |
| 5 | 8 | 16.41 |
| 6 | 16 | 34.78 |
| 7 | 32 | 62.51 |
| 8 | 64 | 132.36 |
| 9 | 128 | 245.49 |
| 10 | 256 | 498.35 |
| TAAENEFVTLK for KRT6C | | |
| **Standard** | **Standard Concentration (fmol/µL)** | **Heavy to Light area ratio** |
| 1 | 0.5 | 9.3 |
| 2 | 2 | 37.87 |
| 3 | 4 | 67.52 |
| 4 | 16 | 239.61 |
| 5 | 32 | 552 |
| 6 | 64 | 1068.04 |
| LTEPADTITDAVK for KLK 1 | | |
| **Standard** | **Standard Concentration (fmol/µL)** | **Heavy to Light area ratio** |
| 1 | 0.5 | 0.10 |
| 2 | 1 | 0.35 |
| 3 | 2 | 0.82 |
| 4 | 4 | 1.88 |
| 5 | 8 | 3.89 |
| 6 | 16 | 7.67 |
| 7 | 32 | 14.25 |
| 8 | 64 | 31.77 |
| 9 | 128 | 67.95 |
| GTNYLADVFEK for S100A7 | | |
| **Standard** | **Standard Concentration (fmol/µL)** | **Heavy to Light area ratio** |
| 1 | 1 | 7.66 |
| 2 | 2 | 30.71 |
| 3 | 4 | 57.06 |
| 4 | 8 | 77.47 |
| 5 | 16 | 248.79 |
| 6 | 32 | 440.72 |
| 7 | 64 | 914.01 |
| QEILAALEK for PSAP | | |
| **Standard** | **Standard Concentration (fmol/µL)** | **Heavy to Light area ratio** |
| 1 | 0.5 | 0.65 |
| 2 | 2 | 2.66 |
| 3 | 4 | 5.87 |
| 4 | 8 | 11.30 |
| 5 | 16 | 21.52 |
| 6 | 32 | 44.65 |
| 7 | 64 | 90.13 |
| 8 | 128 | 192.46 |
| DLQNFLK for S100A9 | | |
| **Standard** | **Standard Concentration (fmol/µL)** | **Heavy to Light area ratio** |
| 1 | 0.5 | 0.14 |
| 2 | 1 | 0.37 |
| 3 | 2 | 0.78 |
| 4 | 4 | 1.45 |
| 5 | 8 | 2.52 |
| 6 | 16 | 4.93 |
| 7 | 32 | 9.39 |
| 8 | 64 | 19.24 |
| 9 | 128 | 41.45 |
| 10 | 256 | 92.66 |
| AALSYVSEIGK for BPIFB2 | | |
| **Standard** | **Standard Concentration (fmol/µL)** | **Heavy to Light area ratio** |
| 1 | 0.5 | 0.79 |
| 2 | 1 | 2.89 |
| 3 | 2 | 5.55 |
| 4 | 4 | 12.21 |
| 5 | 8 | 24.71 |
| 6 | 16 | 44.40 |
| 7 | 32 | 101.30 |
| 8 | 64 | 183.91 |
| 9 | 128 | 399.58 |
| 10 | 256 | 818.02 |
| DAGPLLISLK for CORO1A | | |
| **Standard** | **Standard Concentration (fmol/µL)** | **Heavy to Light area ratio** |
| 1 | 0.5 | 6.78 |
| 2 | 2 | 20.40 |
| 3 | 4 | 50.75 |
| 4 | 8 | 78.18 |
| 5 | 16 | 180.68 |
| 6 | 32 | 408.15 |
| 7 | 64 | 673.42 |
| LQATVQELQK for CORO1A | | |
| **Standard** | **Standard Concentration (fmol/µL)** | **Heavy to Light area ratio** |
| 1 | 0.5 | 5.59 |
| 2 | 1 | 10.54 |
| 3 | 2 | 19.04 |
| 4 | 4 | 37.72 |
| 5 | 8 | 60.48 |
| 6 | 16 | 124.85 |
| 7 | 32 | 247.84 |
| 8 | 64 | 472.59 |
| SILLTEQALAK for LACRT | | |
| **Standard** | **Standard Concentration (fmol/µL)** | **Heavy to Light area ratio** |
| 1 | 0.5 | 0.85 |
| 2 | 1 | 1.98 |
| 3 | 2 | 3.75 |
| 4 | 4 | 7.89 |
| 5 | 8 | 13.75 |
| 6 | 16 | 29.38 |
| 7 | 32 | 56.51 |
| 8 | 64 | 112.66 |
| 9 | 128 | 236.71 |
| 10 | 256 | 474.64 |
| SYPGLTSYLVR for LCN2 | | |
| **Standard** | **Standard Concentration (fmol/µL)** | **Heavy to Light area ratio** |
| 1 | 1 | 0.60 |
| 2 | 2 | 1.85 |
| 3 | 4 | 4.17 |
| 4 | 8 | 10.38 |
| 5 | 16 | 21.36 |
| 6 | 32 | 48.42 |
| 7 | 64 | 93.90 |
| 8 | 128 | 190.75 |
| AGEVQEPELR for AZGP1 | | |
| **Standard** | **Standard Concentration (fmol/µL)** | **Heavy to Light area ratio** |
| 1 | 0.5 | 0.04 |
| 2 | 2 | 0.14 |
| 3 | 4 | 0.27 |
| 4 | 8 | 0.52 |
| 5 | 16 | 1.15 |
| 6 | 32 | 2.07 |
| 7 | 64 | 4.48 |
| 8 | 128 | 9.06 |
| VTVLGQPK for IGLL5 | | |
| **Standard** | **Standard Concentration (fmol/µL)** | **Heavy to Light area ratio** |
| 1 | 0.5 | 0.71 |
| 2 | 2 | 2.51 |
| 3 | 4 | 4.79 |
| 4 | 8 | 8.87 |
| 5 | 16 | 16.50 |
| 6 | 32 | 35.28 |
| 7 | 64 | 64.47 |
| 8 | 128 | 135.06 |

**Figure S1**: **Standard curve obtained for the peptides selected for protein quantification.** Heavy labelled peptide pool at varying concentration was spiked in the saliva digest (at a concentration of 1 µg/µL) obtained by pooling the saliva samples of 25 healthy controls. Concentration of standards used for calibration curve and corresponding heavy to light ratio for each peptide is provided in the supplementary table S6. Correlation coefficient of 0.99 was obtained for all peptide except for LQATVQELQK corresponding to CORO1A. vertical line in the curve corresponds to LOQ. Standard curve for **(a)** FSVVYAK (AHSG), **(b)** TAAENEFVTLK (KRT6C), **(c)** LTEPADTITDAVK (KLK1), **(d)** GTNYLADVFEK (S100A7), **(e)** QEILAALEK (PSAP), **(f)** DLQNFLK (S100A9), **(g)** AALSYVSEIGK (BPIFB2), **(h)** DAGPLLISLK (CORO1A), **(i)** LQATVQELQK (CORO1A), **(j)** VTVLGQPK (IGLL5), **(k)** AGEVQEPELR (AZGP1), **(l)** SILLTEQALAK (LACRT) and **(m)** SYPGLTSYLVR (LCN2).


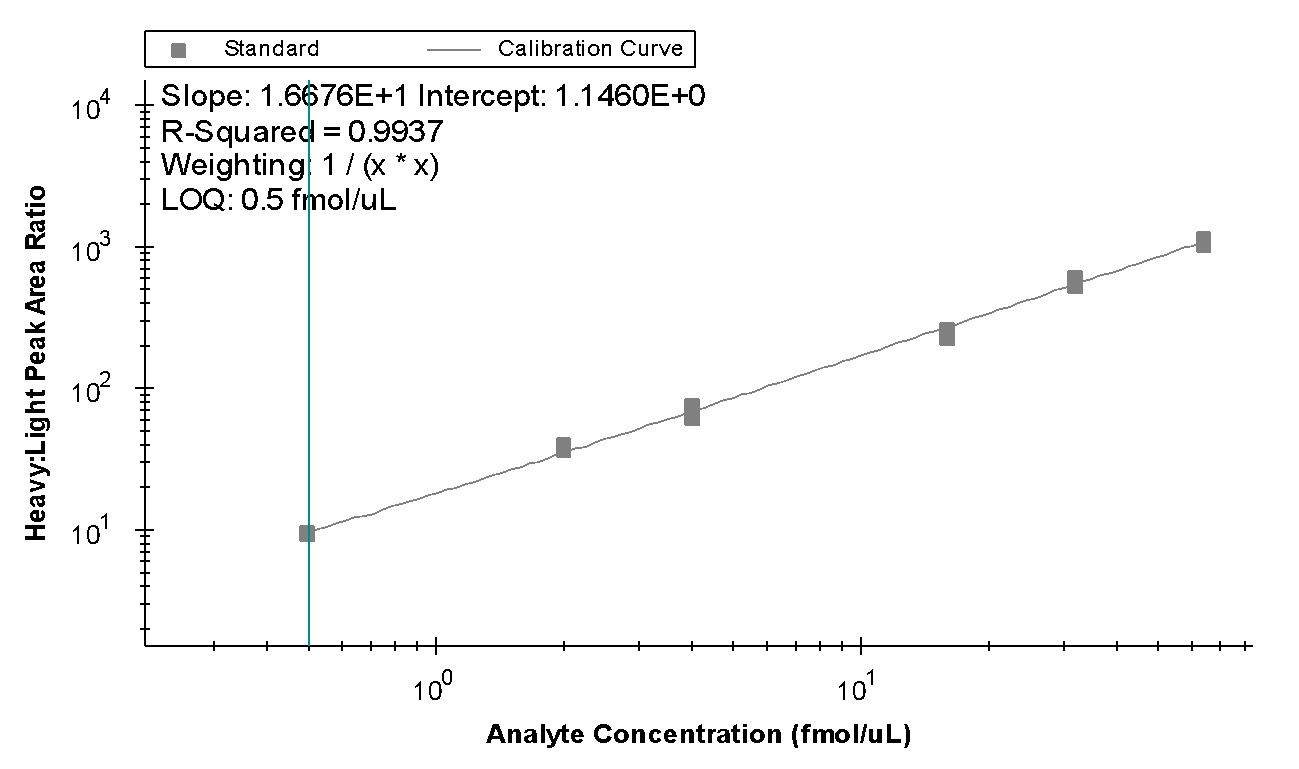


Protein: KRT6C

Peptide: TAAENEFVTLK

**b**


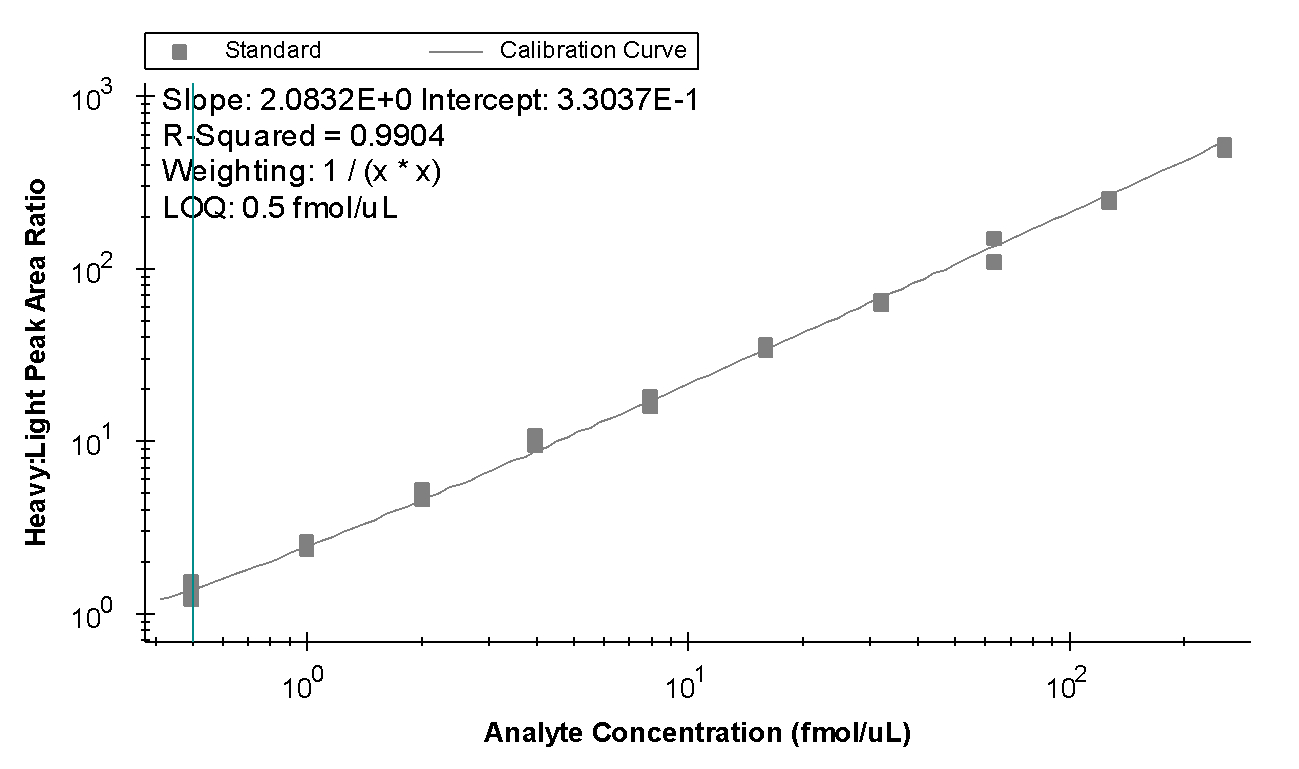


Protein: AHSG

Peptide: FSVVYAK

**a**


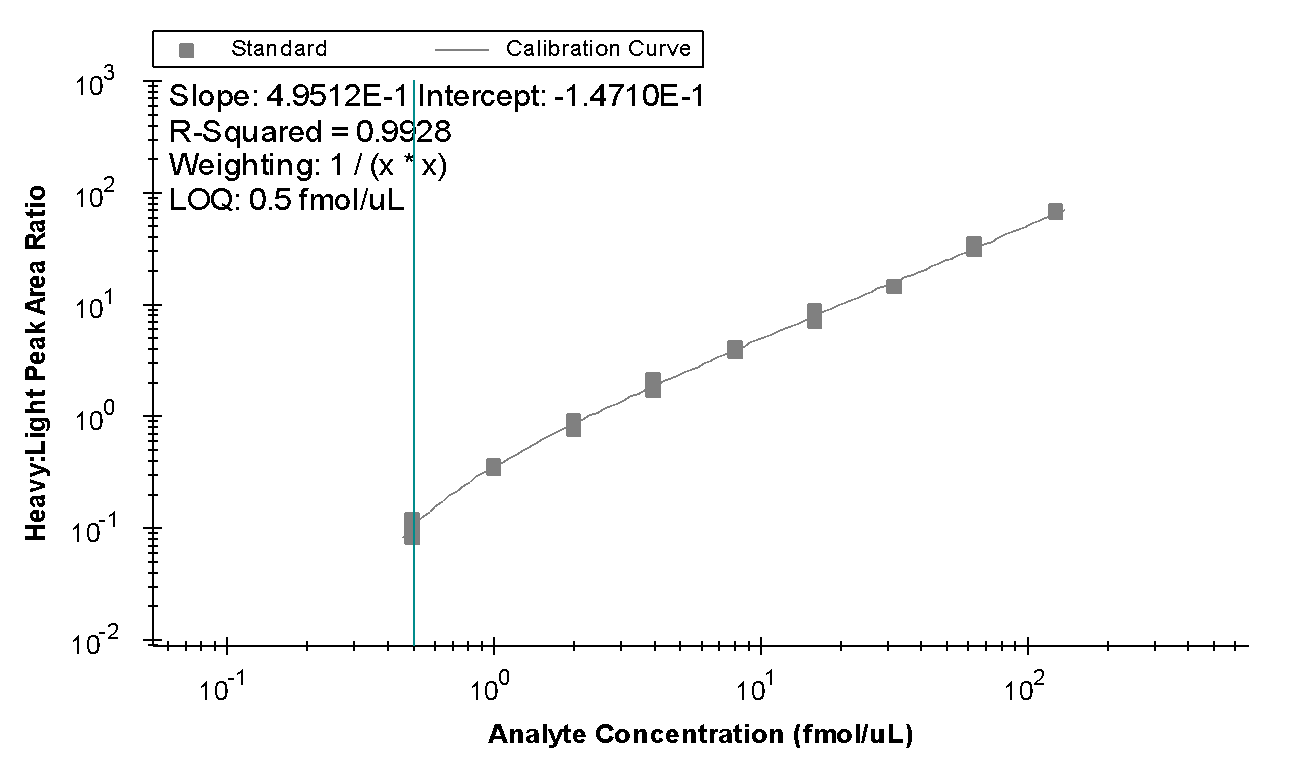


Protein: KLK1

Peptide: LTEPADTITDAVK

**c**


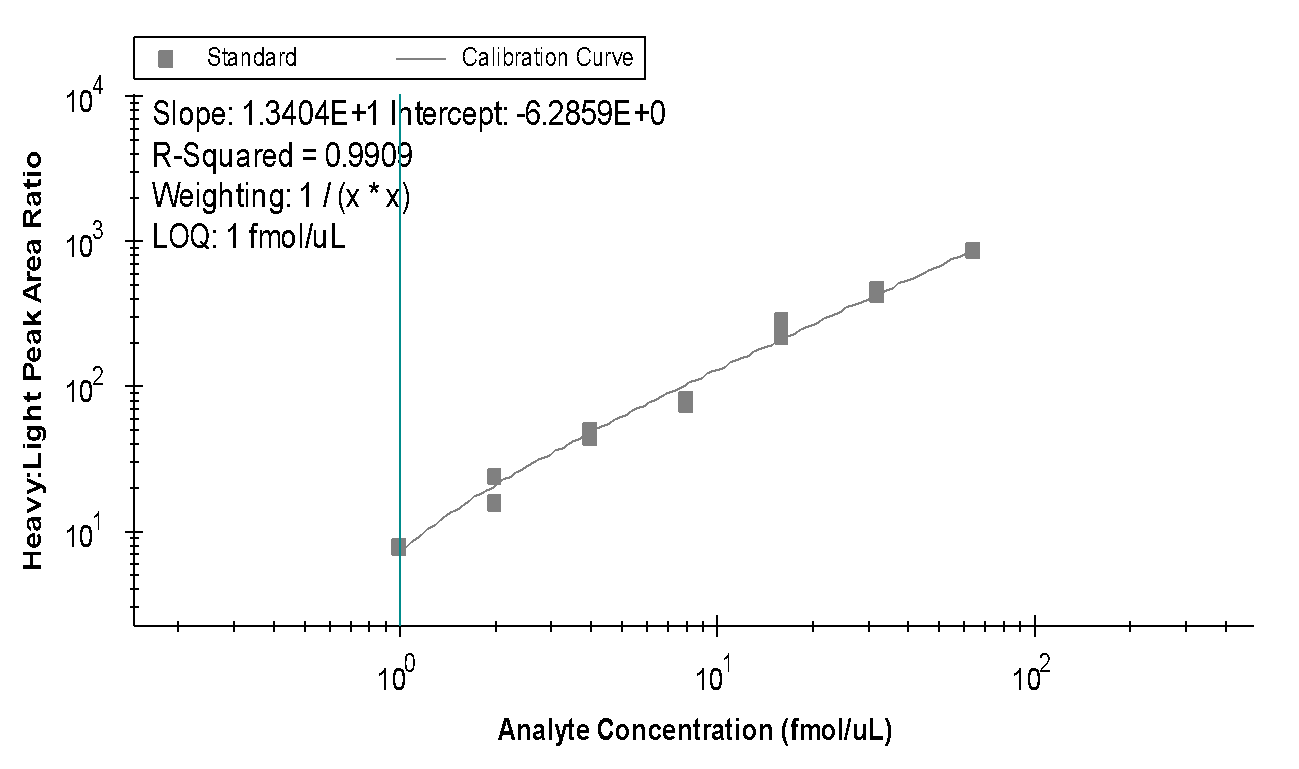


Protein: S100A7

Peptide: GTNYLADVFEK

**d**


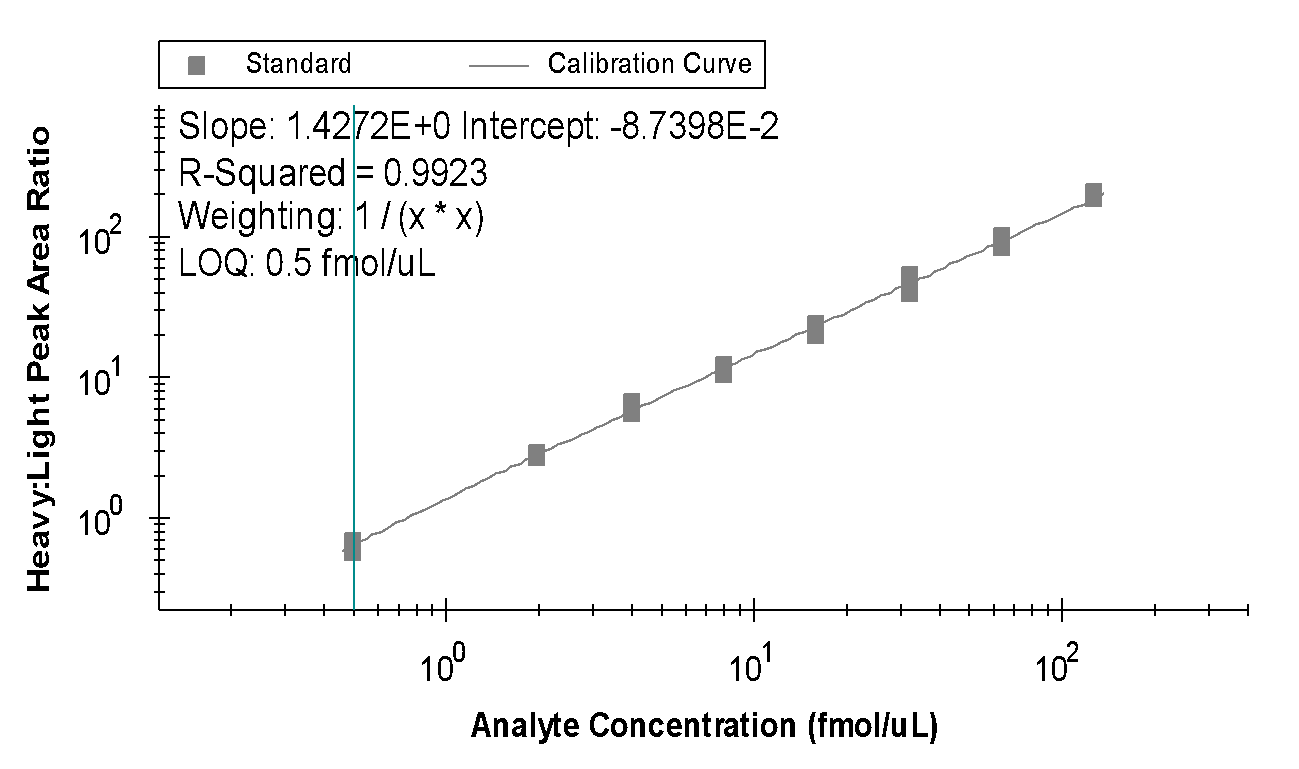


Protein: PSAP

Peptide: QEILAALEK

**e**


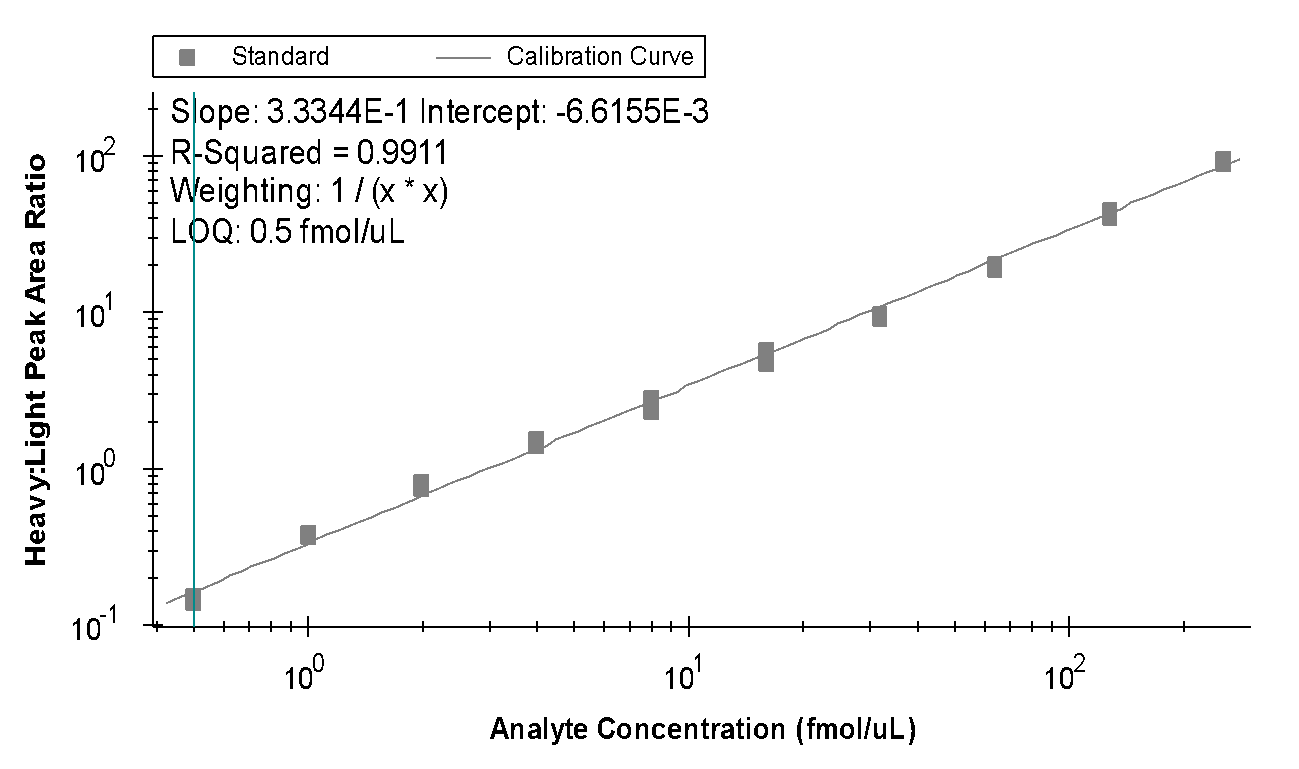


**f**

Protein: S100A9

Peptide: DLQNFLK


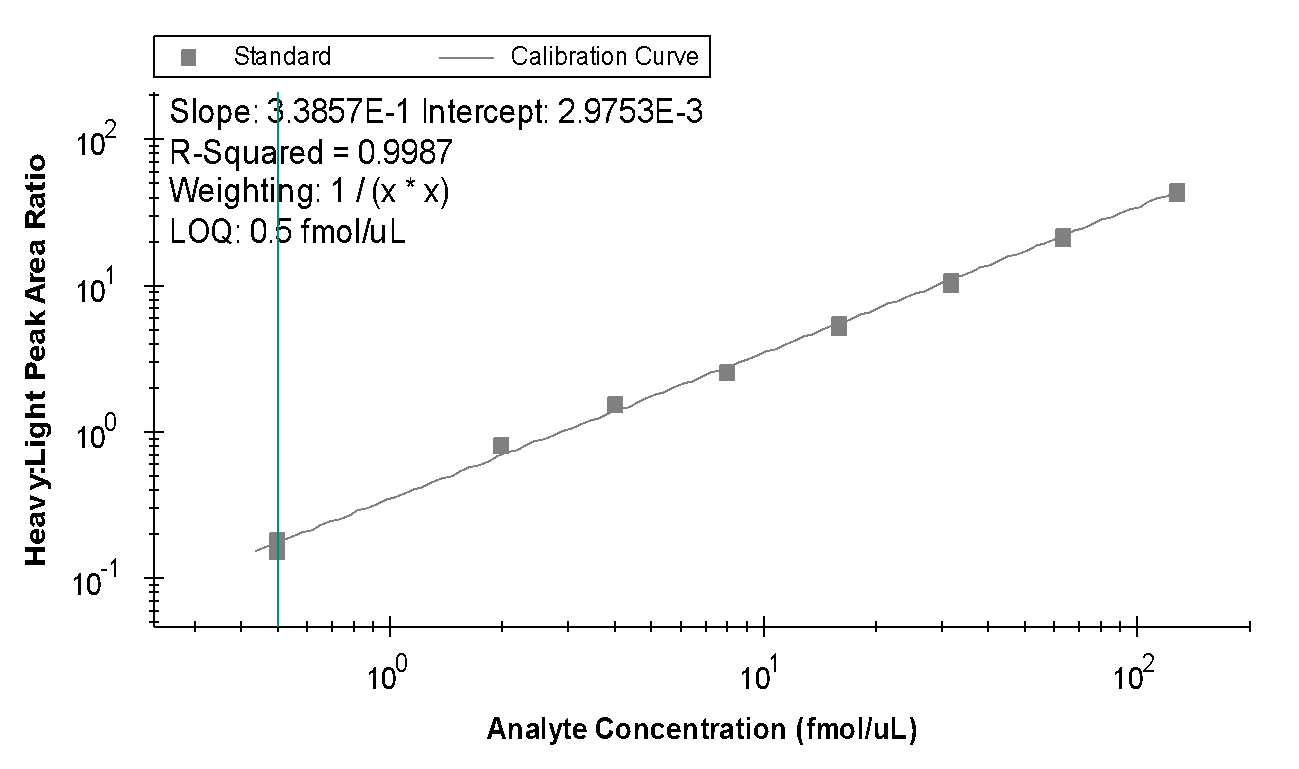

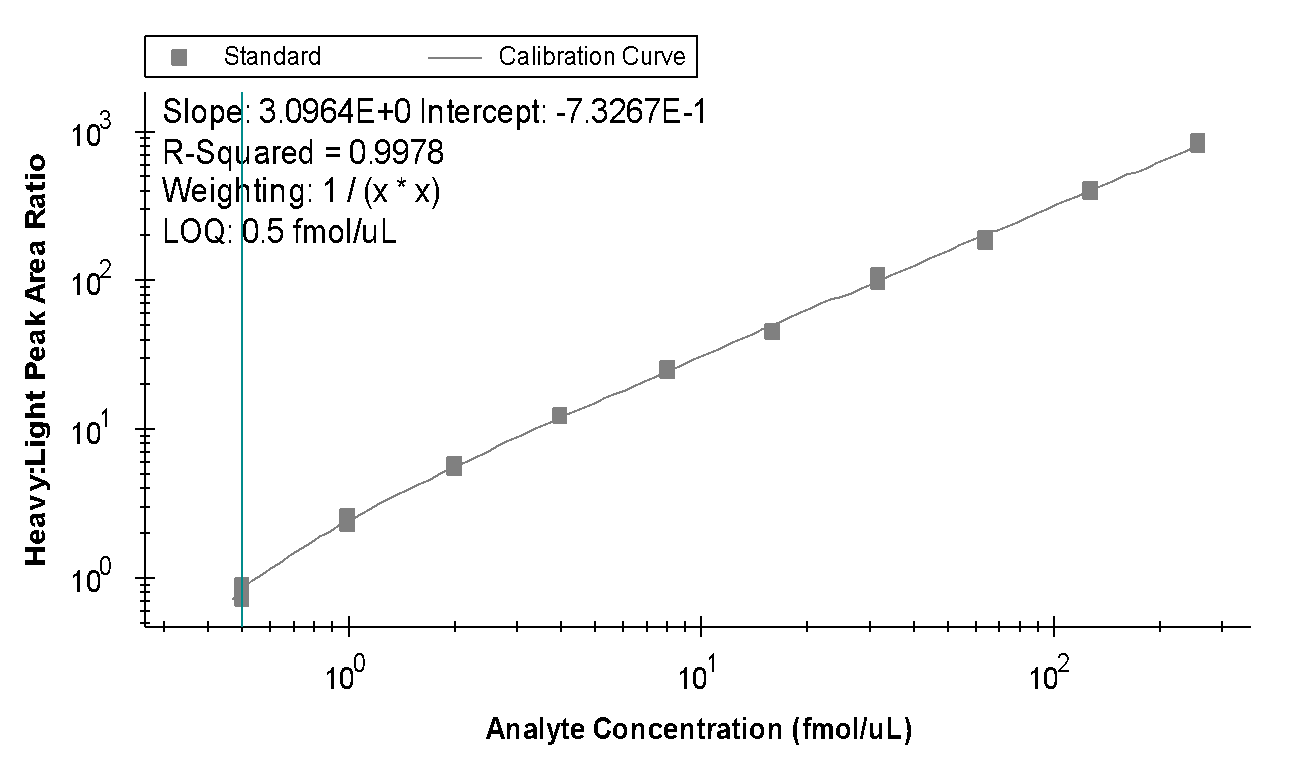


**g**

Protein: BPIFB2

Peptide: AALSYVSEIGK


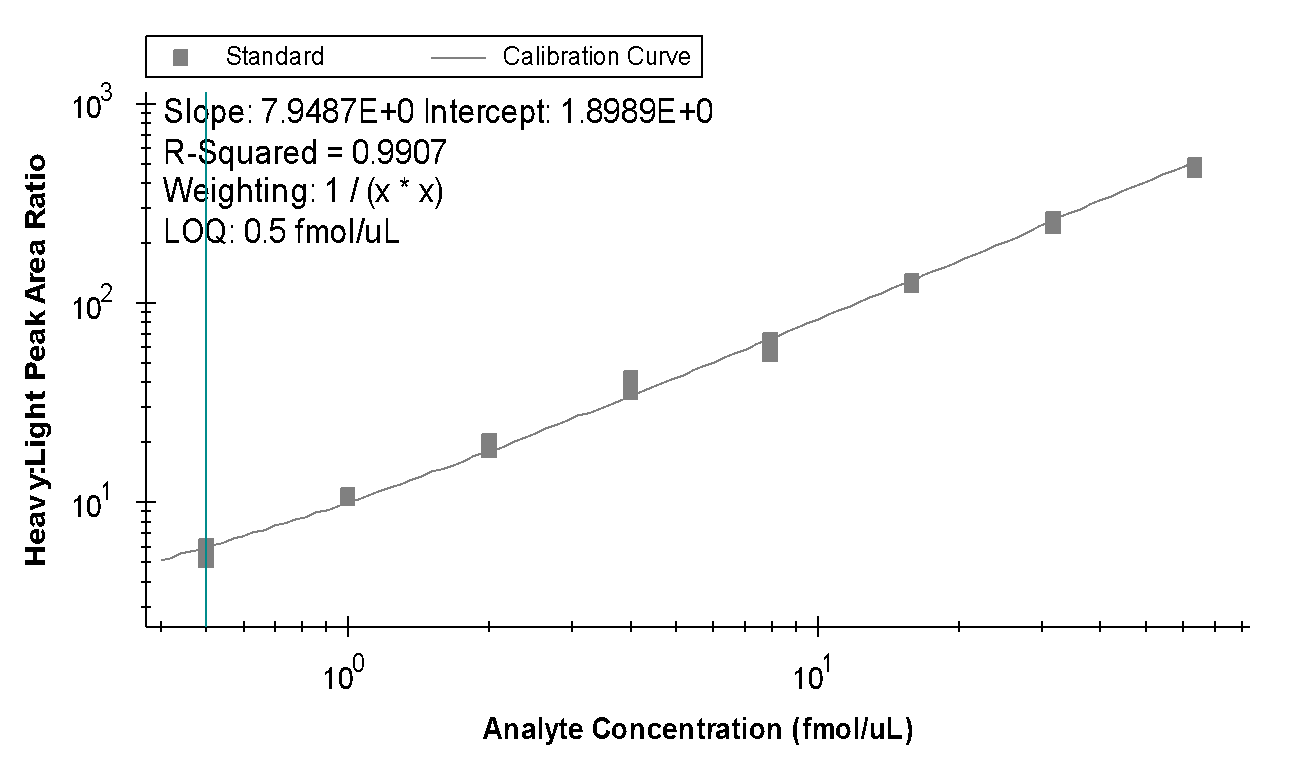


**h**

Protein: CORO1A

Peptide: DAGPLLISLK


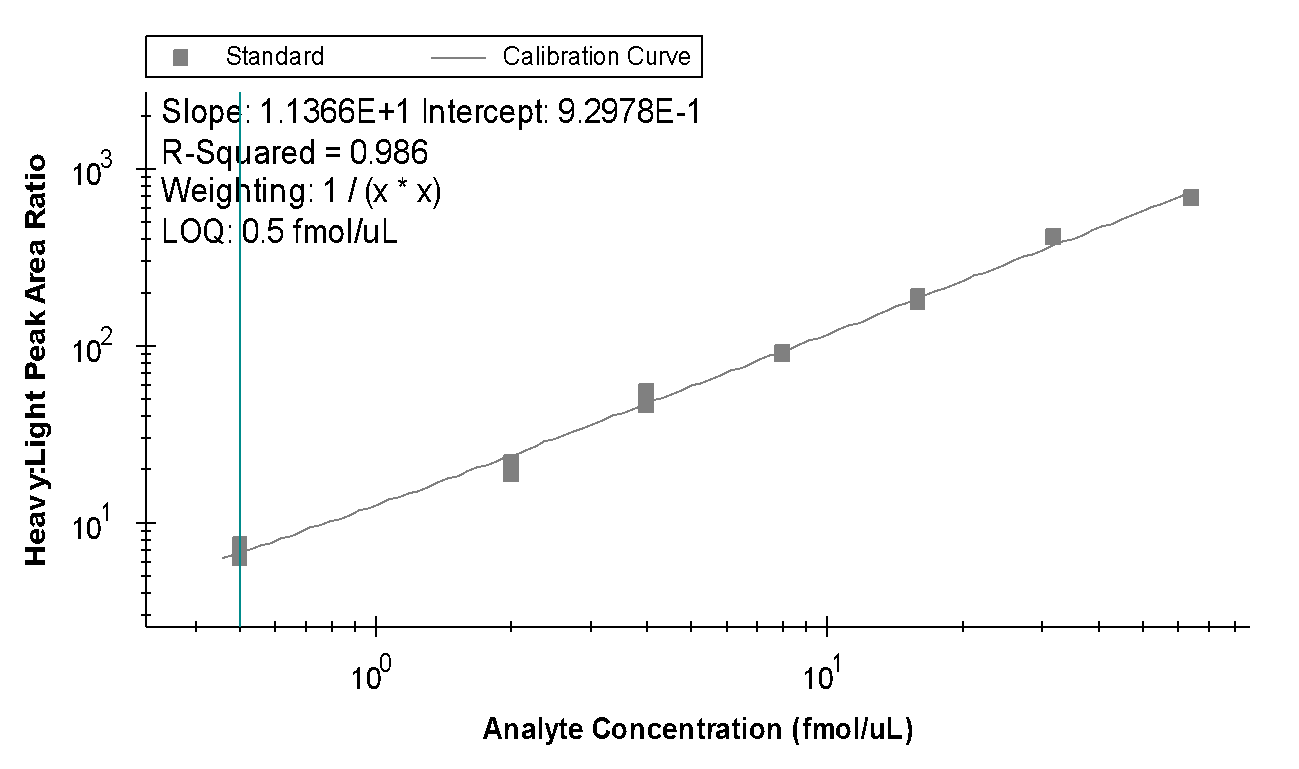


Protein: CORO1A

Peptide: LQATVQELQK

**i**


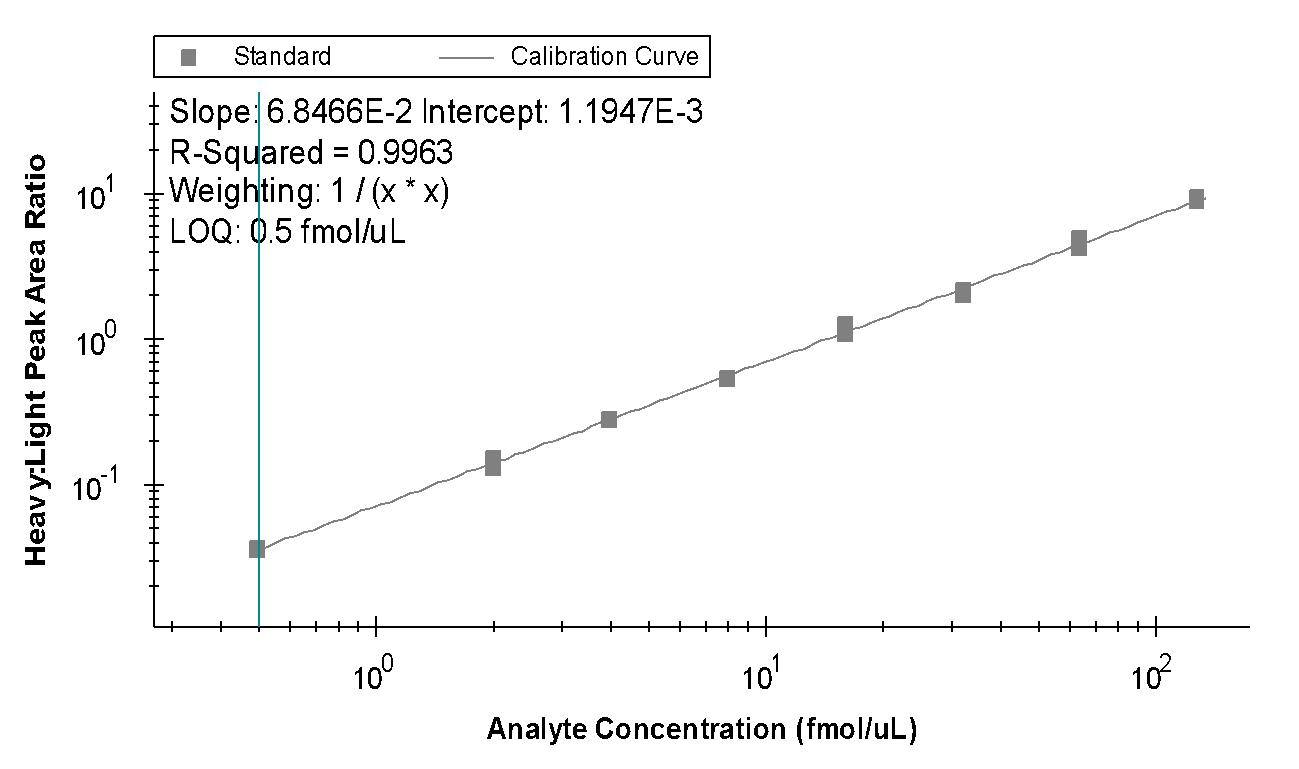


Protein: AZGP1

Peptide: AGEVQEPELR

**k**


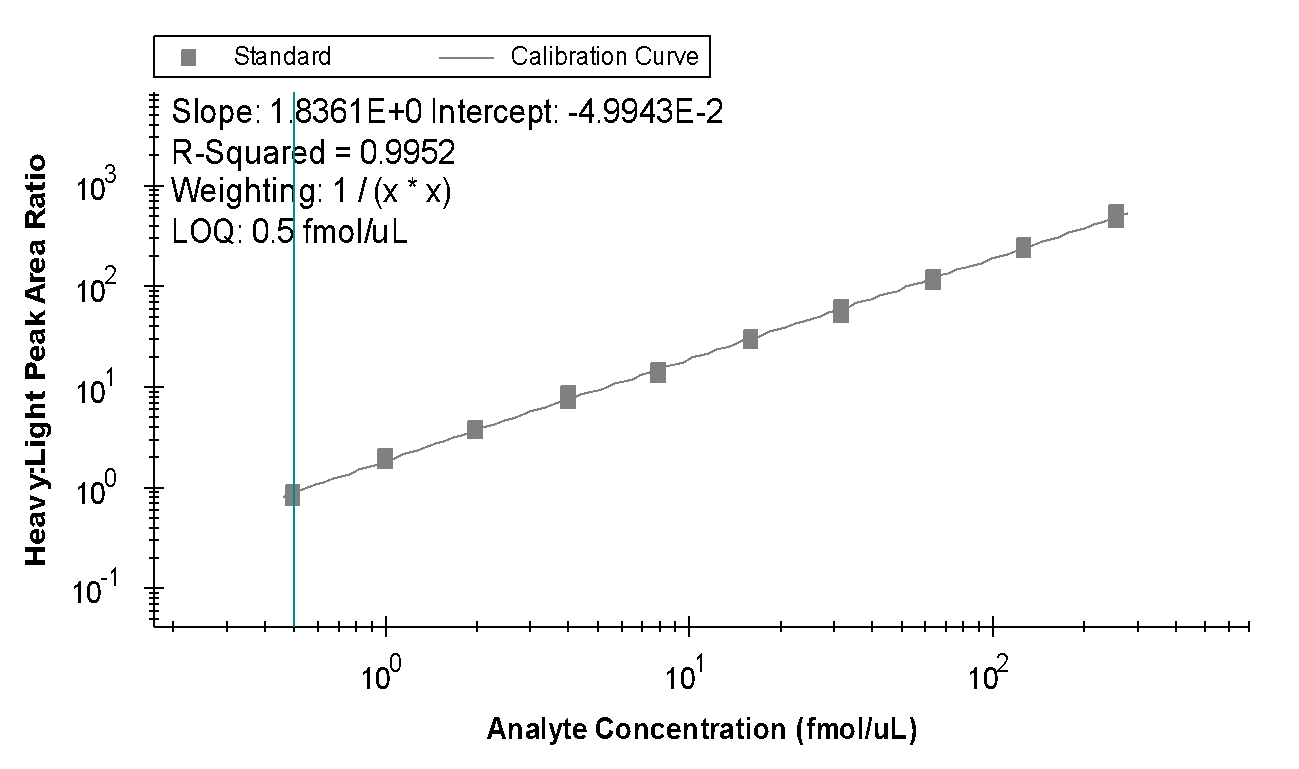


Protein: LACRT

Peptide: SILLTEQALAK

**l**


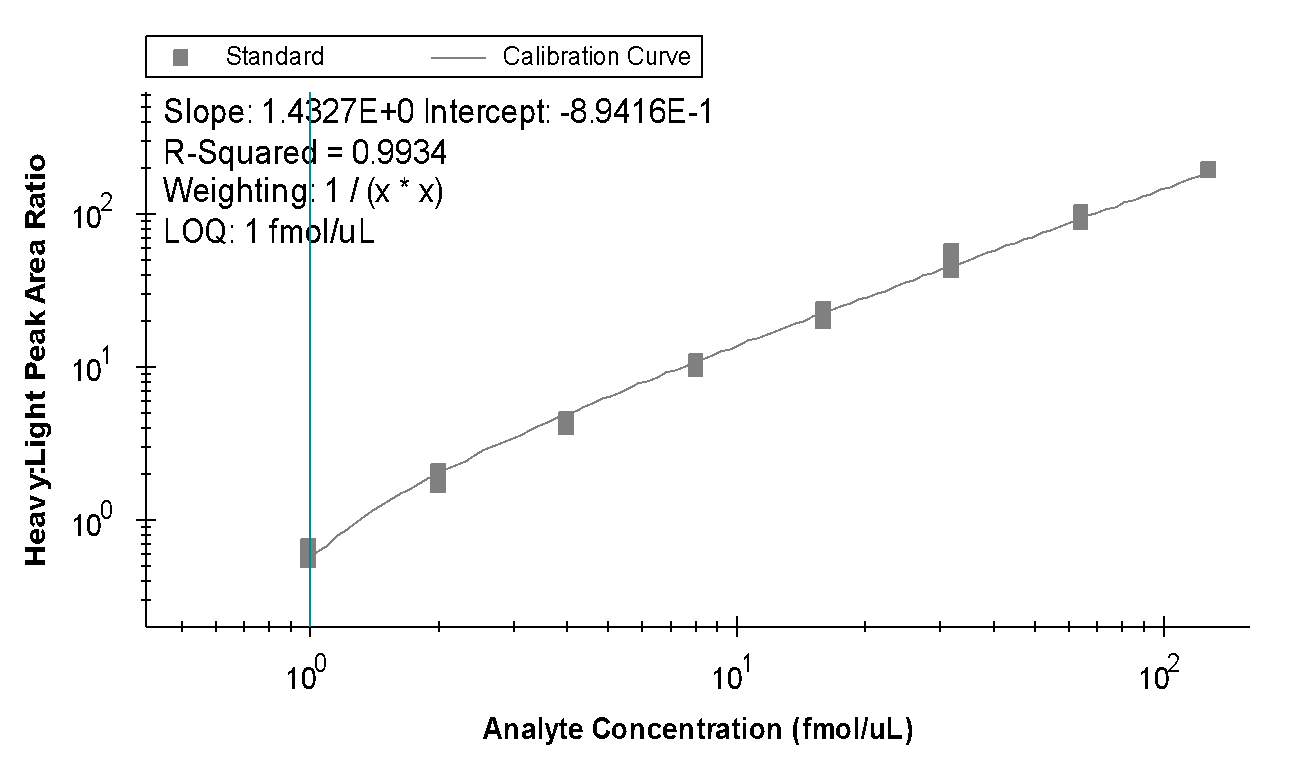


Protein: LCN2

Peptide: SYPGLTSYLVR

**m**

Protein: IGLL5

Peptide: VTVLGQPK

**j**

**Parameters used to determine post treatment disease status as No evidence of disease and residual disease.**

The disease status is determined on every follow-up after the completion of treatment protocol. For this, clinical examination is done which includes indirect laryngoscopy or endoscopy under anaesthesia to observe any abnormal growth. Neck palpation is done to check for lymph node metastasis in neck region and CT scan is done to check for lymph node metastasis in chest region.

If no growth is observed, disease status is characterized as no evidence of disease. However, if any abnormal growth is detected, Fine needle aspiration cytology/biopsy is done to confirm the presence of tumor. If tumor is confirmed, disease status is characterized as residual disease.
